# Relieving DYRK1A repression of MKL1 confers an adult-like phenotype to human infantile megakaryocytes

**DOI:** 10.1172/JCI154839

**Published:** 2022-10-03

**Authors:** Kamaleldin E. Elagib, Ashton Brock, Cara M. Clementelli, Goar Mosoyan, Lorrie L. Delehanty, Ranjit K. Sahu, Alexandra Pacheco-Benichou, Corinne Fruit, Thierry Besson, Stephan W. Morris, Koji Eto, Chintan Jobaliya, Deborah L. French, Paul Gadue, Sandeep Singh, Xinrui Shi, Fujun Qin, Robert Cornelison, Hui Li, Camelia Iancu-Rubin, Adam N. Goldfarb

**Affiliations:** 1 Department of Pathology, University of Virginia School of Medicine, Charlottesville, Virginia, USA.; 2 Tisch Cancer Institute, Department of Hematology and Medical Oncology, Icahn School of Medicine at Mount Sinai, New York, NY.; 3 Normandie University, UNIROUEN, INSA Rouen, CNRS, COBRA UMR 6014, Rouen, France.; 4 HealthChart LLC, Memphis, Tennessee, USA.; 5 Center for iPS Cell Research and Application, Kyoto University, Kyoto, Japan.; 6 Center for Cellular and Molecular Therapeutics and; 7 Department of Pathology and Laboratory Medicine, The Children’s Hospital of Philadelphia, Philadelphia, Pennsylvania, USA.; 8 Academy of Medical Sciences, School of Medicine, Zhengzhou University, Zhengzhou, Henan, China.; 9 Department of Pathology, Molecular and Cell-Based Medicine, Icahn School of Medicine at Mount Sinai, New York, New York, USA.

**Keywords:** Development, Hematology, Bone marrow differentiation

## Abstract

Infantile (fetal and neonatal) megakaryocytes (Mks) have a distinct phenotype consisting of hyperproliferation, limited morphogenesis, and low platelet production capacity. These properties contribute to clinical problems that include thrombocytopenia in neonates, delayed platelet engraftment in recipients of cord blood stem cell transplants, and inefficient ex vivo platelet production from pluripotent stem cell–derived Mks. The infantile phenotype results from deficiency of the actin-regulated coactivator, MKL1, which programs cytoskeletal changes driving morphogenesis. As a strategy to complement this molecular defect, we screened pathways with the potential to affect MKL1 function and found that DYRK1A inhibition dramatically enhanced Mk morphogenesis in vitro and in vivo. Dyrk1 inhibitors rescued enlargement, polyploidization, and thrombopoiesis in human neonatal Mks. Mks derived from induced pluripotent stem cells responded in a similar manner. Progenitors undergoing Dyrk1 inhibition demonstrated filamentous actin assembly, MKL1 nuclear translocation, and modulation of MKL1 target genes. Loss-of-function studies confirmed MKL1 involvement in this morphogenetic pathway. Expression of Ablim2, a stabilizer of filamentous actin, increased with Dyrk1 inhibition, and Ablim2 knockdown abrogated the actin, MKL1, and morphogenetic responses to Dyrk1 inhibition. These results delineate a pharmacologically tractable morphogenetic pathway whose manipulation may alleviate clinical problems associated with the limited thrombopoietic capacity of infantile Mks.

## Graphical abstract

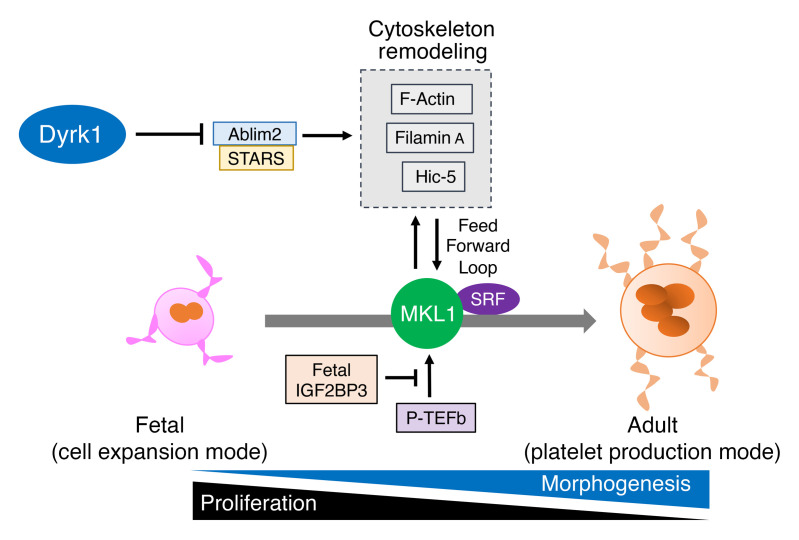

## Introduction

Human megakaryocytes (Mks) undergo dramatic changes during ontogenic progression from fetal through adult stages. These changes include diminishing proliferation and a concomitant increase in adult-type morphogenesis (1–3). Adult-type morphogenesis is comprised of an orchestrated sequence of cellular transitions that enable efficient platelet production. This sequence of polyploidization, enlargement, and proplatelet formation stands apart from the core differentiation program of platelet protein and organelle induction, which is fully active throughout ontogeny (3, 4). The limited morphogenesis potential of infantile (fetal and neonatal) Mks has several clinical consequences, including the high incidence of thrombocytopenia in critically ill neonates and delayed platelet engraftment in transplant patients receiving cord blood (CB) stem cells. Regarding neonatal thrombocytopenia, approximately 30% of newborns in neonatal intensive care units experience thrombocytopenia, the severity of which increases with prematurity; many receive platelet transfusions intended to diminish the risk of intraventricular hemorrhage (1, 5). With allogeneic hematopoietic stem cell transplants, CB recipients experience a significant delay in platelet recovery compared with adult progenitor recipients, leading to increased transfusions as well as increased morbidity (1, 6, 7). Importantly, CB recipients have similar numbers of marrow Mks as adult progenitor recipients, indicating a defect at the level of platelet production efficiency rather than megakaryocytic engraftment (8).

The limited capacity of infantile Mks for ex vivo platelet production also has clinical relevance. Global demographic trends predict worsening shortages of donor platelet units for transfusion, prompting a need for a donor-independent sources derived from cultured Mks (9–12). CB and pluripotent stem cell (PSC) Mks offer the only feasible sources for this application due to their capacity for large-scale expansion (10, 13, 14) and cryopreservation (15, 16). In addition, PSC-derived progenitors may undergo gene editing to produce HLA-null platelets (17), which is of particular importance due to the growing number of allo-sensitized patients requiring HLA-matched platelets (18). However, human PSC-derived Mks phenocopy those derived from the fetal liver in their limited capacity for morphogenesis (19). Thus, a major obstacle to scaling up ex vivo platelet production for clinical use consists of the low efficiency in thrombopoiesis. Development of specialized culture systems to replicate the mechanical and biophysical properties of the marrow milieu has improved platelet productivity (20–23). However, major limitations remain due to scalability, cost effectiveness, limited platelet shelf life, and safety standards.

Within the process of morphogenesis, endomitosis leading to polyploidization plays a central role in the determination of platelet production capacity. Prior observations suggest that a single mature Mk after 3 rounds of endomitosis (16N) can yield approximately 125-fold more platelets than 8 mature Mks produced by 3 standard mitoses (2N) (2). In adults, Mks can increase their polyploidization as part of the marrow response to increased platelet demand; the lack of such a capability in infants, on top of their lower basal ploidy, underlies their propensity to develop thrombocytopenia in response to a variety of stressors (1, 2). Hyperproliferation and impaired polyploidization also predispose infantile Mks to neoplastic transformation, explaining the occurrence of unique megakaryoblastic leukemias that appear only in early childhood (1, 24, 25).

Mechanisms governing ontogenic changes in Mk morphogenesis remain ill-defined. Their molecular definition, at a basic level, will illuminate cellular events guiding the process of polyploidization. At a translational level, these mechanisms will provide targets for the treatment of thrombocytopenias associated with infantile Mks as well as approaches for the scale-up of ex vivo platelet production. Along these lines, recent studies have implicated the onco-fetal RNA-binding protein IGF2BP3 as a driver of the infantile phenotype, acting to restrain morphogenesis through the repression of megakaryoblastic leukemia 1 levels (MKL1, also known as MRTFA) (26, 27). MKL1 functions as a coactivator of the serum response factor (SRF) transcription factor in programming the differentiation of multiple cell types, including multiple muscle and hematopoietic lineages. It exerts these functions through the control of genes involved in actin cytoskeleton remodeling and is, in turn, regulated by the cytoskeletal status of monomeric G-actin, or globular actin, which prevents its nuclear localization (28). MKL1 plays a critical role in driving adult Mk morphogenesis and undergoes upregulation and nuclear translocation in response to the megakaryopoietic cytokine thrombopoietin (TPO) (28–30).

Experiments herein identify an approach to bypass the morphogenetic blockade imposed by IGF2BP3 in infantile Mks, applying a strategy of MKL1 potentiation. In the screening of MKL1 regulatory pathways, we found that inhibition of dual-specificity tyrosine-regulated kinase (Dyrk) uniquely exerted a phenotypic and molecular rescue that (a) elicited adult-type morphogenesis in neonatal and induced PSC (iPSC-derived) Mk progenitors, (b) enhanced neonatal platelet production both in vitro and in vivo, and (c) induced the sustained nuclear translocation of MKL1 associated with upregulation of MKL1/SRF target factors via actin signaling. Multiple approaches indicated specific involvement of the DYRK1A isoform, which has been linked to Mk leukemogenesis in Down syndrome (31). A critical downstream effector in this pathway was identified as the F-actin, or filamentous actin, stabilizer actin-binding LIM protein 2 (Ablim2), with levels that were controlled by Dyrk kinase activity and knockdown that abrogated the response to Dyrk inhibition. These results thus identify molecular circuitry in Mk morphogenesis that participates in ontogenic programming and offering a potential target for therapeutic intervention.

## Results

### The morphogenesis blockade in infantile Mks is abrogated by Dyrk kinase inhibition.

**Figure 1:**
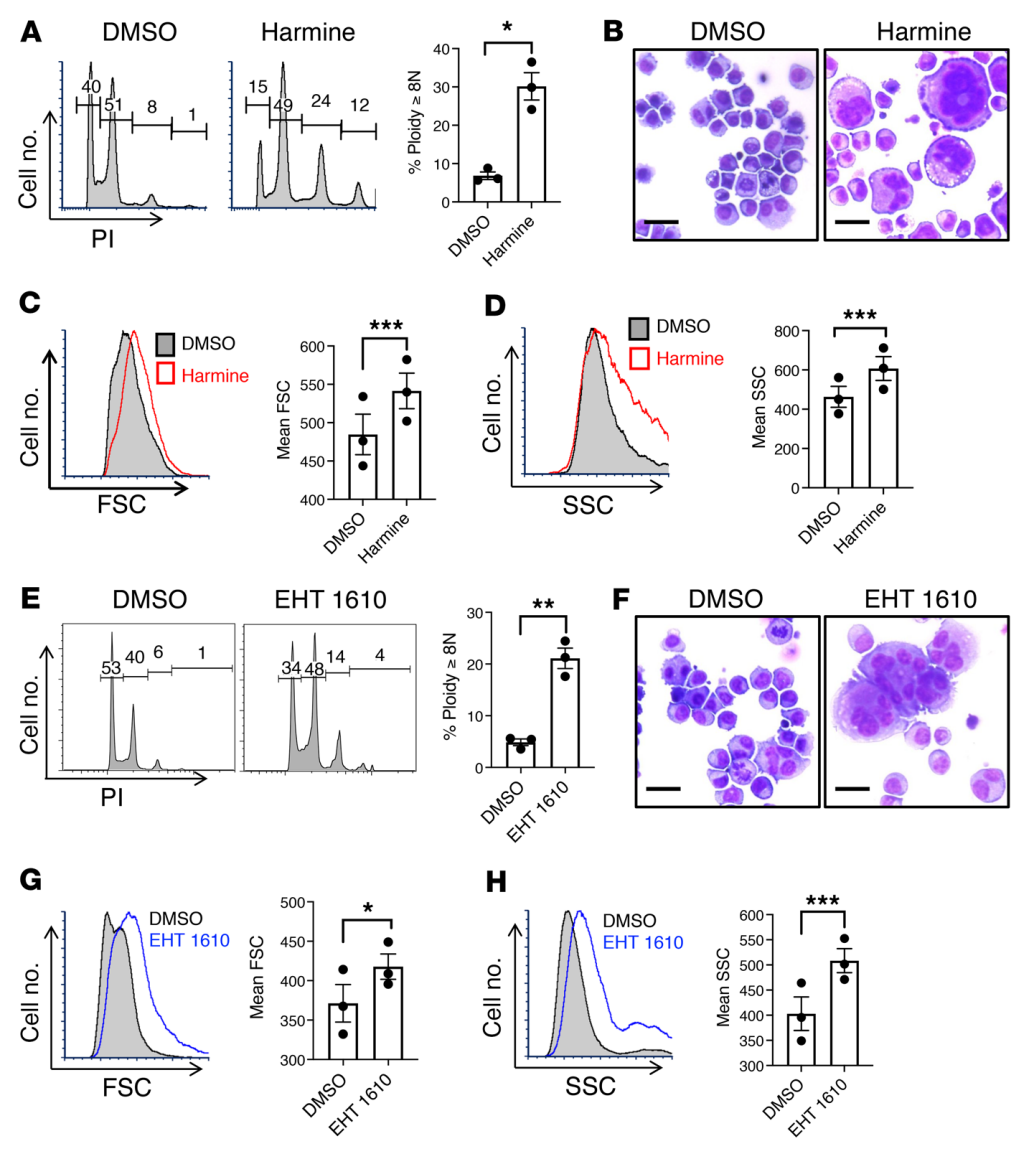
Induction of adult-type morphogenesis in neonatal Mks treated with Dyrk kinase inhibitors. (**A**–**D**) Umbilical cord blood CD34^+^ cells were cultured in Mk medium with or without 5 μM Dyrk inhibitor harmine for 6 days. Cells were analyzed either by flow cytometry after costaining with FITC-anti-CD41 and PI, or by microscopy of cytospins. (**A**) Mk polyploidization (PI staining). (**B**) Morphology of cytospins subjected to Wright stain and light microscopy, original magnification, ×200; scale bar: 20 μm. (**C**) Mk size (FSC). (**D**) Mk complexity/granulation (SSC). Graphs for **A**–**D**, mean ± SEM for 3 independent experiments, Student’s *t* test. (**E**–**H**) Cells cultured and analyzed as in **A** but with or without 5 μM selective Dyrk1a inhibitor EHT 1610. (**E**) Mk polyploidization (PI). (**F**) Morphology in cytospins as in **B**. (**G**) Mk size (FSC). (**H**) Mk complexity/granulation (SSC). Graphs for **E**–**H**, mean ± SEM for 3 independent experiments. *P < 0.05; **P < 0.01; ***P < 0.005, Student’s *t* test.

Deficient MKL1 expression contributes to the impaired morphogenesis of infantile Mks (26). To determine whether a strategy of functional enhancement could compensate for this deficiency, neonatal progenitors consisting of umbilical CB CD34^+^ cells underwent screening with inhibitors of kinases reported to restrain MKL1 activity (32–36). These experiments revealed that Dyrk kinase inhibition using harmine, a potent Dyrk inhibitor, uniquely and strongly enhanced morphogenesis, as reflected by induction of polyploidization (propidium iodide [PI]), enlargement (forward scatter [FSC]), cytoplasmic complexity (side scatter [SSC]), and morphologic features (Figure 1, A–D, and Supplemental Figure 1, A and B; supplemental material available online with this article; https://doi.org/10.1172/JCI154839DS1). Treatment with this inhibitor had no effect on megakaryocytic commitment, as reflected by the percentage of CD41^+^ cells, but did diminish the overall number of cells approximately 2-fold (Supplemental Figure 1C). Harmine also suppressed an infantile Mk feature: leaky expression of the erythroid marker glycophorin A (GPA) (26) (Supplemental Figure 1D). Analysis of a panel of compounds structurally related to harmine (37, 38) supported Dyrk1 kinases as the relevant target, rather than the nonkinase target monoamine oxidase (Supplemental Figure 2, A and B). A broad kinome screen has shown that harmine’s targets are highly restricted, with significant inhibitory activity at 10 μM toward 5 related kinases (DYRK1A, DYRK1B, DYRK2, CLK1, and CLK2) (39). Nevertheless, to minimize the potential for off-target effects, we also subjected neonatal progenitors to additional Dyrk1 inhibitors EHT 1610 and FC 162, which have structures and binding modes completely unrelated to harmine (40, 41). Notably, EHT 1610 demonstrates exquisite specificity for Dyrk1, with a 10-fold higher IC_50_ for Dyrk2 (40). Accordingly, EHT 1610 and FC 162 enhanced morphogenesis in infantile Mks in a similar manner to harmine (Figure 1, E–H, and Supplemental Figure 2, C and D) and consistent with Dyrk1 as the likely target. As with the harmine treatment, EHT did not affect the percentage of CD41^+^ cells but decreased the overall number approximately 2-fold (Supplemental Figure 2C).

**Figure 2:**
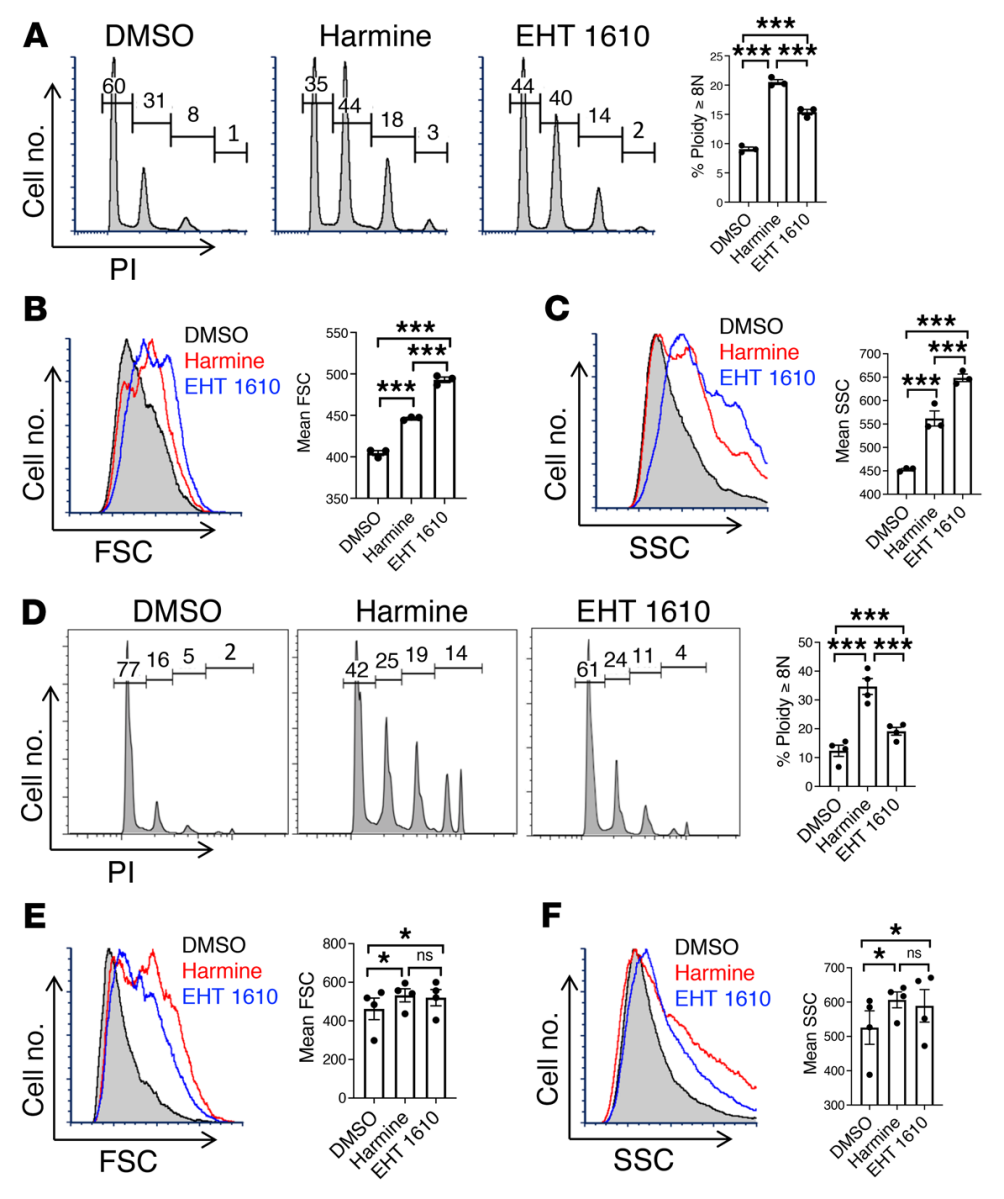
Dyrk1 inhibitors elicit adult-type morphogenesis in iPSC-derived Mks. (**A**–**C**) Human iPSC-derived Mk progenitors cultured 5 days in SDF differentiation medium with or without 2.5 μM inhibitors (harmine or EHT 1610) underwent flow cytometry after costaining with FITC-anti-CD41 and PI. (**A**) Mk polyploidization (PI). (**B**) Mk size (FSC). (**C**) Mk complexity/granulation (SSC). Graphs for **A**–**C**, mean with or without SEM for 3 independent experiments. ***P < 0.005, 1-way ANOVA with Tukey’s post hoc test. (**D**–**F**) Conditionally immortalized imMKCL cells cultured 6 days in doxycycline-free differentiation medium with or without 5 μM inhibitors were analyzed as in **A**–**C**. (**D**) Mk polyploidization (PI). (**E**) Mk size (FSC). (**F**) Mk complexity/granulation (SSC). Graphs for **D**–**F**, mean ± SEM for 3 independent experiments. *P < 0.05; ***P < 0.005; 1-way ANOVA with Tukey’s post hoc test.

The morphogenetic effects of Dyrk1 inhibition on primary neonatal progenitors raised the possibility of extending this approach to additional, clinically applicable systems for ex vivo megakaryopoiesis wherein morphogenesis is limited. Human PSC-derived Mks correspond in ontogenic stage to fetal liver progenitors and have minimal capacity for morphogenesis, even less than neonatal progenitors (19). The amenability to personalization and gene editing, however, has made iPSCs a particularly desirable source for the development of donor-independent platelets (17, 42). We therefore tested the effects of Dyrk1 inhibition on iPSC-derived Mks, generated as previously described (16). In this system, both harmine and EHT 1610 markedly augmented several parameters of morphogenesis, attaining polyploidization levels typical of adult Mks (Figure 2, A–C, and Supplemental Figure 3A). Recently, conditional immortalization of iPSC-derived megakaryocytic progenitors has facilitated their large-scale expansion while maintaining the potential for inducible platelet production (22, 43). The prototypic cell line derived in this manner, imMKCL, showed limited morphogenesis upon induction of differentiation, but potently responded to Dyrk1 inhibition with attainment of adult-type levels of polyploidization (Figure 2, D–F, and Supplemental Figure 3B). Efficient ex vivo progenitor expansion has also been accomplished by treating neonatal CD34^+^ cells with the aryl hydrocarbon antagonist SR1 (44, 45). As expected, SR1-treated CB progenitors underwent extensive self-renewal, with CD34 retained on 80% of cells after 8 days of expansion culture. When transferred into megakaryocytic differentiation medium, these cells demonstrated a near-complete morphogenesis blockade. This blockade was effectively reversed by Dyrk1 inhibitors (Supplemental Figure 4, A–E), supporting the applicability of this approach in several distinct scalable culture systems with the potential for donor-independent platelet production.

### Dyrk kinase inhibition enhances platelet production in infantile Mks.

**Figure 3:**
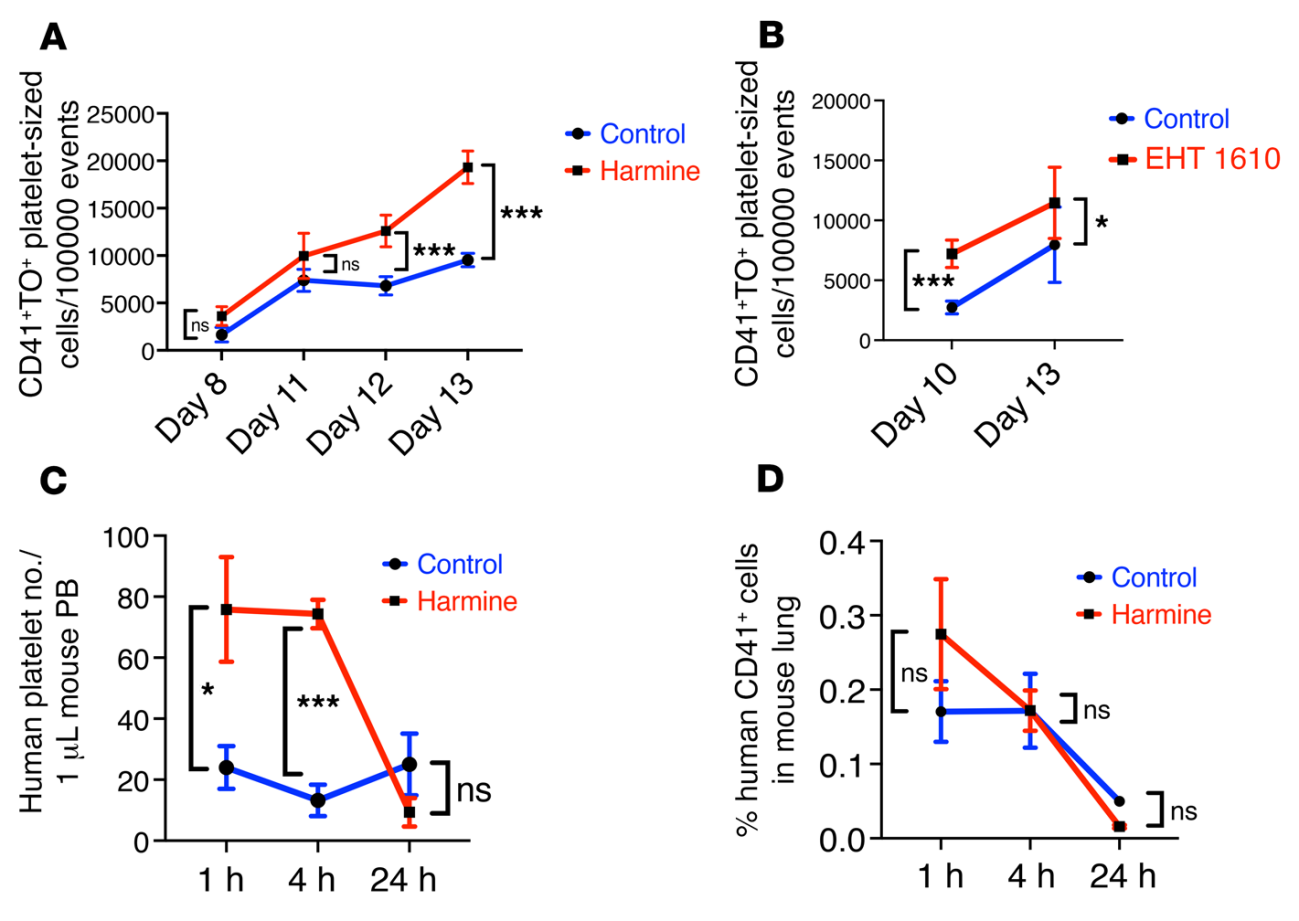
Enhanced platelet production by neonatal Mks treated with Dyrk1 inhibitors. (**A** and **B**) In vitro platelet release assay. Cord blood CD34^+^ cells were cultured for up to 13 days in Mk medium with or without 5 μM harmine or 2.5 μM EHT 1610. Culture supernatants underwent flow cytometry after labeling with APC-anti-CD41 and thiazole orange (TO). Gating was based on size and CD41^+^/TO^+^ characteristics of normal donor platelets. Graphs show mean platelet numbers ± SEM for 4 independent experiments. *P < 0.05; ***P < 0.005, Student’s *t* test. (**C**) In vivo platelet release. Cord blood CD34^+^ progenitors were cultured for 11 days in Mk medium with or without 2.5 μM harmine. 4 × 10^6^ cells were transplanted per irradiated NSG mouse. Peripheral blood samples were then evaluated for the presence of human platelets by flow cytometry with a human-specific CD41 antibody. The graph shows circulating human platelet count ± SEM, *n =* 4–6/group. *P < 0.05; ***P < 0.005, 1-way ANOVA with Tukey’s post hoc test. (**D**) Lung entrapment of human mKs. Lung tissue was evaluated for the presence of human mKs by flow cytometry with a human-specific CD41 antibody. The graph shows the percent of cells expressing human CD41 ± SEM, *n =* 4–6/group, 1-way ANOVA with Tukey’s post hoc test.

Efficiency of thrombopoiesis, i.e., platelet production, correlates directly with morphogenetic capacity (2, 46). To examine the effects of Dyrk1 inhibition on neonatal thrombopoiesis, we first conducted in vitro platelet release assays, as previously described (26). These assays demonstrated significant enhancement of platelet release by both inhibitors, harmine and EHT 1610, from cord blood-derived Mks (Figure 3, A and B; Mk numbers in Supplemental Figure 5, A and B), correlating with the effects seen on morphogenesis. The resulting platelets demonstrated appropriate ultrastructural characteristics and agonist responsiveness (Supplemental Figure 5, C and D). For assessment of in vivo thrombopoiesis, our recently developed xenotransplantation assay was employed (15). Mks derived from neonatal progenitors cultured for 11 days in megakaryocytic media with or without 2.5 μM harmine were injected by tail vein at 4 × 10^6^ cells/mouse. At the time of injection, the samples had similar levels of purity (81%–84% CD41^+^) and maturity (42%–49% CD42^+^ and CD41^+^). Quantitation of human platelets in the peripheral blood (PB) over 24 hours showed markedly enhanced in vivo platelet release by the harmine-treated Mks at 1 hour and 4 hours (Figure 3C). Prior studies have shown that intravenous injection of human Mks into immunodeficient mice leads to their retention in pulmonary capillaries, where platelet production then takes place (10). In the current experiments, flow cytometry on lung tissue for human Mks demonstrated uniformity of entrapment between the groups at 1 and 4 hours, supporting that harmine acts through enhancing platelet release (Figure 3D). To address whether in vivo platelet life span was affected by the ex vivo culture conditions, platelets released in cultures of control versus harmine-treated Mks were infused into immunodeficient mice. Flow cytometric quantitation of human platelets showed no significant differences in the rate of decline based on prior ex vivo treatment (Supplemental Figure 5E).

### Implication of the DYRK1A isoform in Mk morphogenesis.

**Figure 4:**
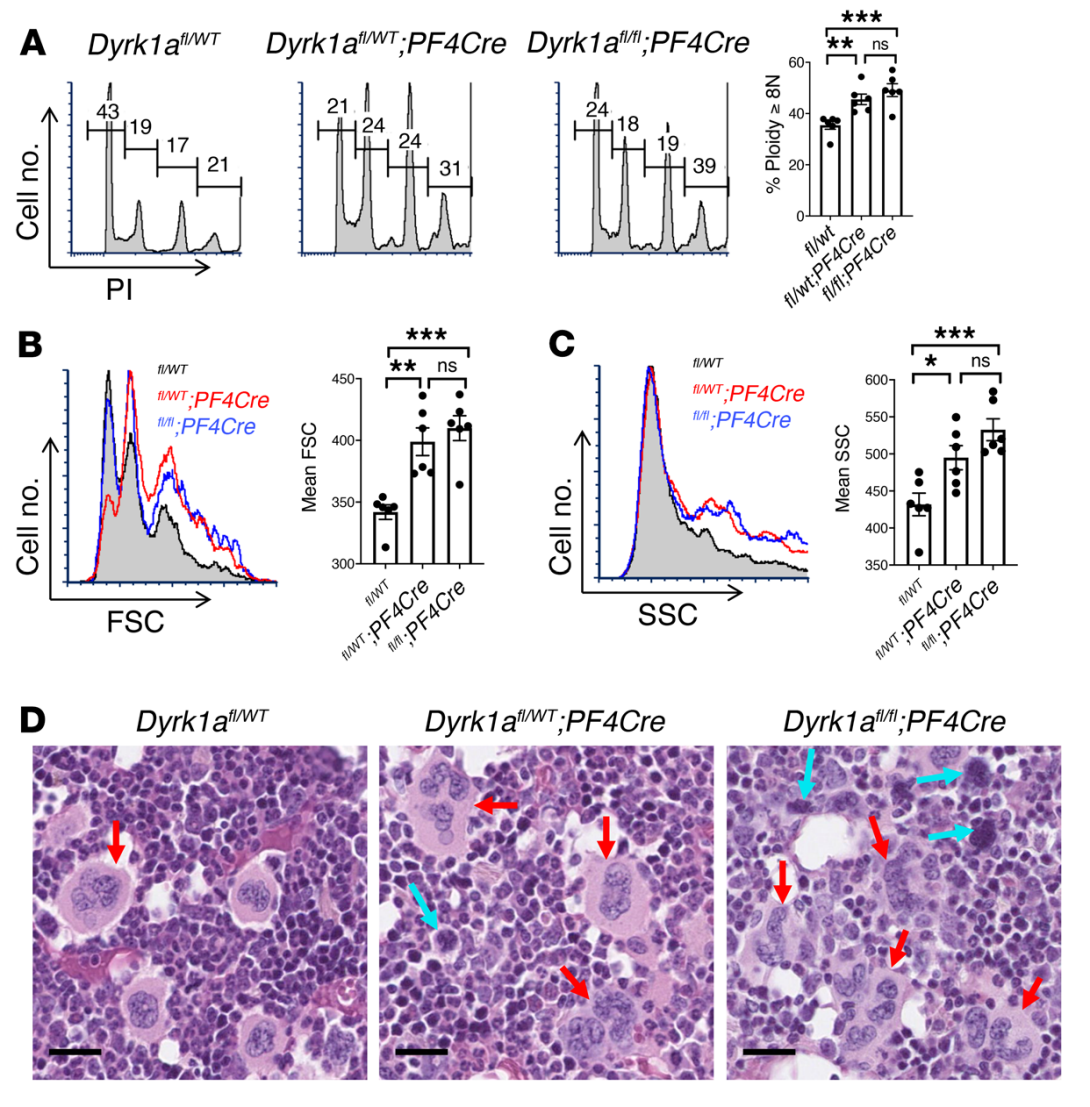
Implication of Dyrk1a isoform in Mk morphogenesis. (**A**–**C**) Marrow samples from indicated murine strains underwent flow cytometry after costaining with FITC-anti-CD41 and PI. (**A**) Mk polyploidization (PI). (**B**) Mk size (FSC). (**C**) Mk complexity/granulation (SSC). Graphs show mean ± SEM, *n =* 6/group, *P < 0.05; **P < 0.01; ***P < 0.005, 1-way ANOVA with Tukey’s post hoc test. (**D**) Representative Mk morphology in H&E-stained marrow sections from indicated strains. Red arrows indicate large polyploid Mk. Blue arrows indicate small pyknotic Mk, original magnification, ×200; scale bar: 20 μm.

Among the 5 Dyrk kinase isoforms, DYRK1A has previously been linked to megakaryopoiesis, its overexpression in Down syndrome contributing to the megakaryoblastic proliferations associated with trisomy 21 (31). To examine the influence of DYRK1A on Mk morphogenesis in vivo, we generated mice with lineage-selective deficiency by crossing *Dyrk1a^fl/fl^* (47) and *Pf4^Cre^* strains (48). Generation of either haploinsufficiency or homozygous deletion enhanced polyploidization, size, and cytoplasmic complexity in marrow Mks (Figure 4, A–C). Histologic evaluation confirmed these changes in the haploinsufficient *Pf4^Cre^*;*Dyrk1a^fl/wt^* mice (Figure 4D, red arrows). In the homozygous *Pf4^Cre^*;*Dyrk1a^fl/fl^* mice, frequent pyknotic Mks also occurred (Figure 4D, blue arrows), consistent with Dyrk1a contribution to cell survival (37). Haplo insufficiency did not alter platelet counts or mean platelet volume (MPV), possibly due to homeostatic compensations in vivo; homozygous deletion decreased platelets and increased MPV, possibly reflecting toxic effects (Supplemental Figure 6, A and B). Neither manipulation affected marrow Mk frequency (Supplemental Figure 6C).

In primary human progenitors, neonatal Mks had significantly higher expression of DYRK1A than adult cells (Supplemental Figure 7A). Retroviral enforcement of DYRK1A in the adult progenitors impaired morphogenesis (ploidy and size) as well as platelet release (Supplemental Figure 7, B–E). Loss-of-function studies could not employ shRNA-mediated knockdown due to the early growth arrest associated with DYRK1A repression, consistent with antiproliferative effects seen with pharmacologic inhibitors (Supplemental Figure 1C and Supplemental Figure 2C). However, the progenitors did tolerate a dominant-negative approach, with retroviral enforcement of the kinase-dead mutant DYRK1A K188R, enhancing morphogenesis in adult and neonatal Mks (Supplemental Figure 7, B–D, F and G). Thus, several approaches support the involvement of the DYRK1A isoform in Mk morphogenesis.

### MKL1 mediates Dyrk1 control of Mk morphogenesis.

**Figure 5:**
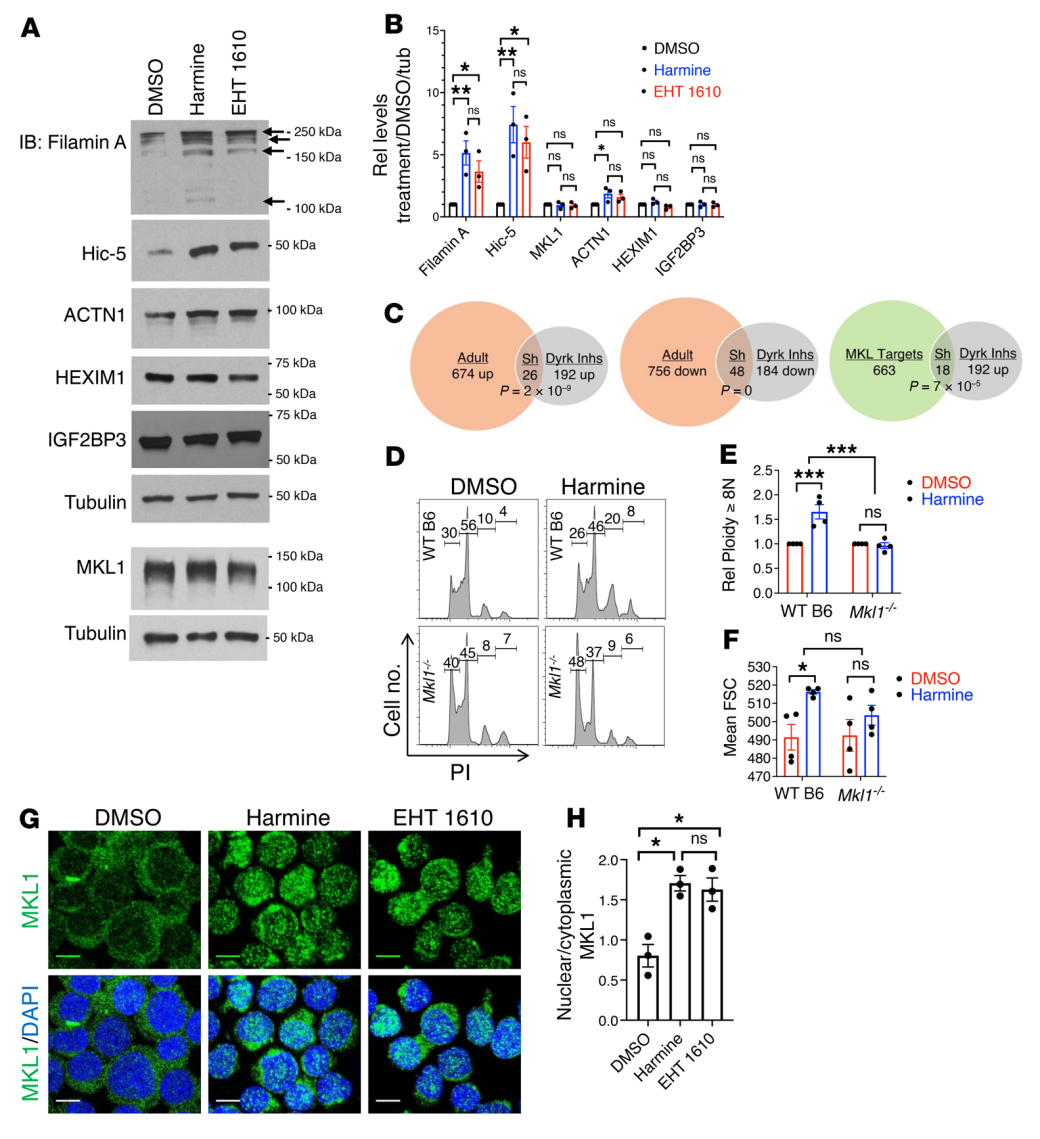
MKL1 involvement in Dyrk control of Mk morphogenesis. (**A**) Effects of Dyrk1 inhibition on targets of MKL1 and P-TEFb. Cord blood CD34^+^ cells cultured for 6 days in Mk medium with or without 5 μM inhibitors underwent immunoblot (IB) of whole cell lysates. Arrows indicate Filamin A isoforms. (**B**) Tubulin-normalized densitometry signals from IBs as in (**A**). Graph shows mean fold changes with inhibitors ± SEM for 3 independent experiments. *P < 0.05; **P < 0.01, 1-way ANOVA with Tukey’s post hoc test. (**C**) Transcriptomic effects in human mK precursors of ontogenic stage (adult versus neonatal) and Dyrk inhibition (neonatal with or without inhibitors). CD34^+^ cells cultured for 4 days in Mk medium underwent purification of CD61^+^ cells followed by RNA-Seq. Overlapping genes (Sh) with hypergeometric *P* values are shown; *n =* 3 independent experiments. See Supplemental Table 1 for gene lists. (**D**–**F**) MKL1 requirement for morphogenesis enhancement. Marrow progenitors from indicated strains cultured for 3 days in murine Mk medium with or without 5 μM harmine underwent flow cytometry after costaining with FITC-anti-CD41 and PI. (**D**) Mk polyploidization (PI). (**E**) Graph shows relative percent Mk ≥ 8N ± SEM, *n =* 4/group. ***P < 0.005, 2-way ANOVA. (**F)** Mk size (FSC). Graph shows mean ± SEM, *n =* 4/group. *P < 0.05, 2-way ANOVA. (**G**) MKL1 localization. Human CD34^+^ progenitors cultured 24 hours in Mk medium with or without 5 μM inhibitors underwent immunofluorescent staining (IF) and confocal microscopy (Zeiss LSM700, original magnification, ×630; scale bar: 10 μm). (**H**) Graph shows mean ratio nuclear/cytoplasmic MKL1 signal ± SEM for 3 independent experiments. *P < 0.05, 1-way ANOVA with Tukey’s post hoc test.

We initially addressed a potential role for MKL1 downstream of Dyrk1 by monitoring the expression of 2 morphogenetic factors induced during adult — but not fetal — megakaryocytic development (26). Both factors, Filamin A and Hic-5, are encoded by MKL1-target genes (49–51). Immunoblotting showed significant enhancement of Filamin A and Hic-5 levels in neonatal Mks subjected to Dyrk inhibition (Figure 5, A and B), with similar responses observed in the iPSC-derived imMKCL line (Supplemental Figure 8A). These experiments demonstrated no effect of Dyrk inhibition on the upstream components of the ontogenic signaling pathway controlling MKL1 (26), P-TEFb, and IGF2BP3 (Figure 5, A and B). P-TEFb activity in these experiments is reported by the levels of its target, HEXIM1 (52–54), which increases in the physiologic fetal-adult Mk transition (26). To gain a broader picture of transcript alterations, purified early-stage Mks underwent RNA-Seq analysis. Genes differentially expressed between adult and neonatal progenitors, i.e., ontogenic genes, were compared with genes affected by Dyrk inhibition, the latter defined as genes similarly regulated by harmine and EHT 1610 (Supplemental Figure 8B). Application of a hypergeometric distribution to quantitate enrichment confirmed significant overlap between ontogenic and Dyrk-related genes, both for upregulation and downregulation; in addition, genes upregulated by Dyrk inhibitors significantly overlapped with canonical MKL1 targets (55) (Figure 5C; see Supplemental Table 1 for gene lists with overlap in red). Gene set enrichment analysis by the Enrichr program (56) was also applied to the genes similarly influenced by ontogenic stage and Dyrk inhibitors. This approach identified actin-related features as the principal gene ontology (GO) categories enriched among the upregulated factors (Table 1). Of note, actin homeostasis has been designated as a conserved, core function of MKL1 (57). Among the commonly downregulated factors, early erythroid genes were identified as a leading category (Supplemental Table 2). Examining gene expression in more mature Mks (day 11), RNA-Seq revealed *ACTB*, which encodes β-actin, as a dominant gene upregulated by harmine (Supplemental Figure 8C), consistent with the notion of MKL1 and actin engaged in a feed-forward loop (28).

**Figure 6:**
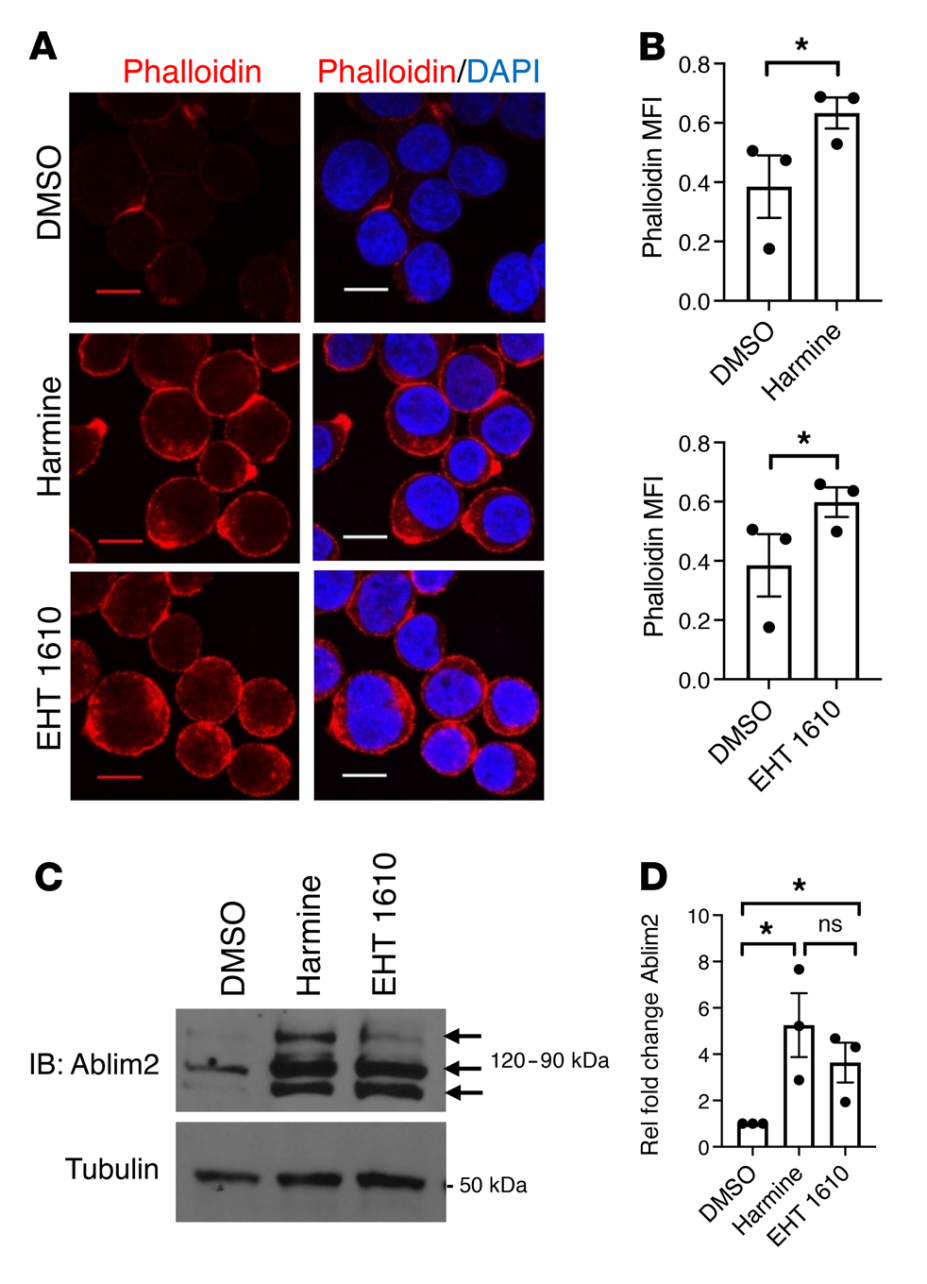
Changes in actin cytoskeleton and actin-associated factors in association with Dyrk1 inhibition. (**A**) Induction of F-actin. Human CD34^+^ progenitors cultured 24 hours in mK medium with or without 5 μM inhibitors underwent staining with indicated fluorescent dyes followed by confocal microscopy original magnification, ×630; scale bar: 10 μm. (**B**) Graphs show mean fluorescent intensity (MFI) of Phalloidin Alexa 594 signals ± SEM for 3 independent experiments as in **A**. *P < 0.05, Student’s *t* test. (**C**) Induction of Ablim2. Progenitors cultured 5 days as in **A** underwent IB of whole cell lysates. Arrows show Ablim2 isoforms. (**D**) Tubulin-normalized densitometry signals from IBs as in (**C**). Graph shows mean fold changes with inhibitors ± SEM for 3 independent experiments. *P < 0.05, 1-way ANOVA with Tukey’s post hoc test.

**Figure 7:**
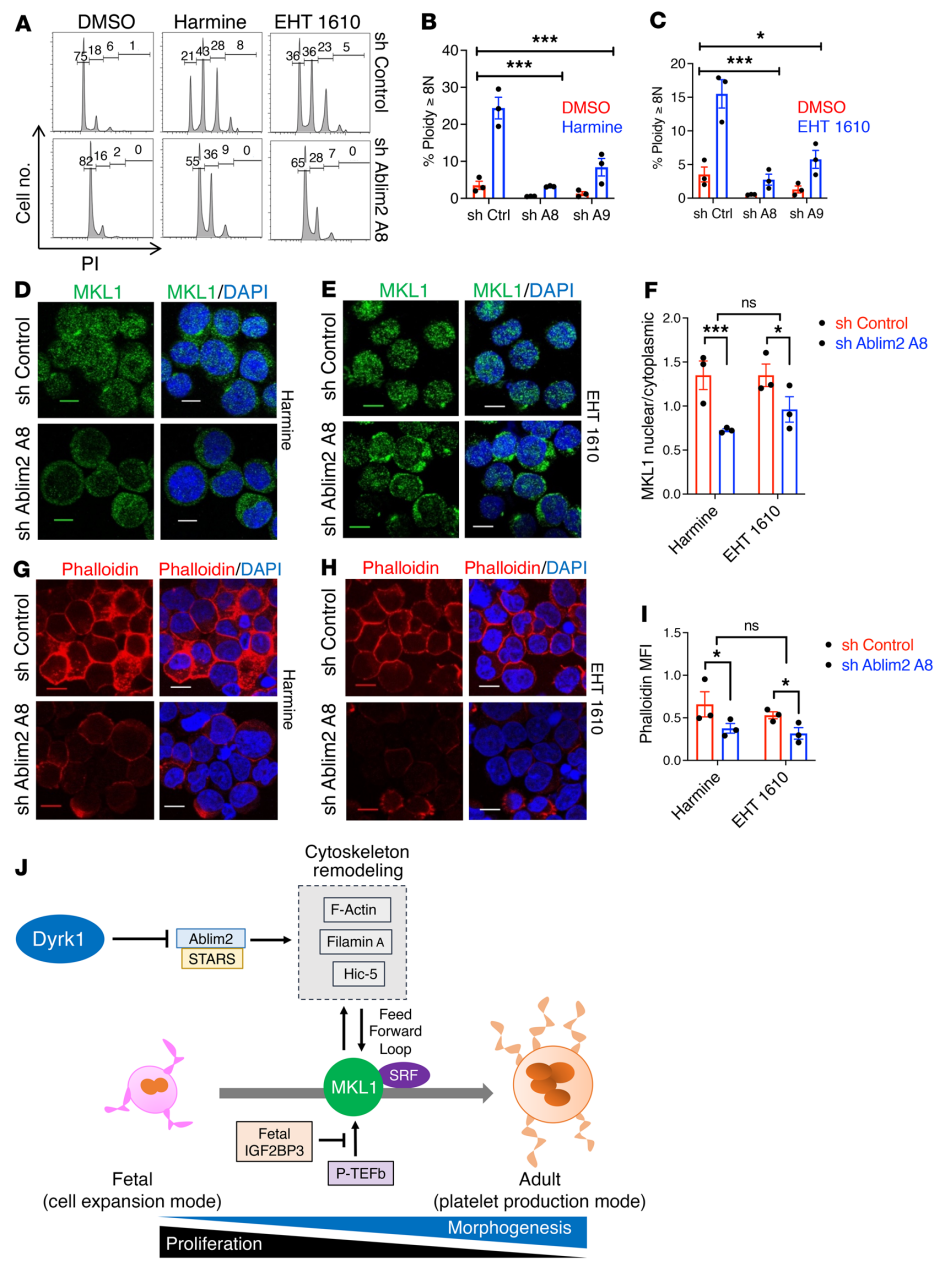
Ablim2 is a critical upstream element in Mk morphogenesis signaling. (**A**) Effect of Ablim2 deficiency on polyploidization response. Neonatal CD34^+^ cells transduced with control or *ABLIM2* targeting lentiviral shRNA constructs were cultured for 5 days in Mk medium with or without 5 μM inhibitors followed by flow cytometry as in Figure 1A. (**B** and **C**) Graphs show mean percent Mk ≥ 8N ± SEM for 3 independent experiments. *P < 0.05; ***P < 0.005, 2-way ANOVA comparing fold induction. (**D**–**F**) MKL1 localization. Cells transduced as in (**A**) underwent 24 hours in Mk medium with or without 5 μM inhibitors followed by IF staining and confocal microscopy. Original magnification, ×630; scale bar: 10 μm. (**F**) Graph shows mean ratio nuclear to cytoplasmic MKL1 signal ± SEM for 3 independent experiments. *P < 0.05; ***P < 0.005, 2-way ANOVA comparing fold change. (**G**–**I**) Induction of F-Actin. Cells as in **D** underwent staining with fluorescent dyes and confocal microscopy. Original magnification, ×630; scale bar: 10 μm. (**I**) Graph shows MFI of Phalloidin Alexa 594 signal ± SEM for 3 independent experiments. *P < 0.05, 2-way ANOVA comparing fold change. (**J**) Diagram of pathways influencing MKL1 in its programming of the fetal-adult mK transition. positive transcription elongation factor (P-TEFb) signaling determines levels of MKL1, and actin cytoskeleton determines function. Diagram shows the influence exerted on MKL1/SRF by Dyrk1 destabilization of the F-actin-binding factors Ablim2/STARS.

To determine the role of MKL1 in the morphogenetic effects of Dyrk inhibition, we cultured marrow progenitors from WT and *Mkl1*^–/–^ mice in megakaryocytic medium with or without harmine. In these conditions, progenitors lacking MKL1 specifically lost the capacity for induction of polyploidization or enlargement, confirming its essential role in responsiveness to Dyrk inhibition (Figure 5, D–F). Because Dyrk inhibitors did not affect the overall levels of MKL1 (Figure 5, A and B), experiments addressed their effects on MKL1 activity, which is controlled at the level of subcellular localization (58). Both Dyrk inhibitors induced a major redistribution of MKL1 from predominantly cytoplasmic to predominantly nuclear, observable in primary progenitors and imMKCL cells through immunofluorescence (IF) (Figure 5, G and H and Supplemental Figure 8D). This effect was also discernable by biochemical fractionation (Supplemental Figure 8E). Note that in the primary progenitors, MKL1 localization and actin status (depicted in Figure 5G, Figure 6A, and Figure 7, D, E, G, and H)) were analyzed at 24 hours, prior to morphogenesis, which was analyzed at 5 days. The rationale for this early analysis was to study early, upstream signaling events that could trigger morphogenesis, rather than later events that might occur secondary to morphogenesis.

### Actin cytoskeletal remodeling is associated with Dyrk1 inhibition.

MKL1 activity depends on the status of the actin cytoskeleton. Monomeric G-actin engages N-terminal RPEL motifs in MKL1 and retains it in an inactive state in the cytoplasm; signaling through RhoA induces MKL1’s nuclear translocation and activation by redistributing the actin into polymeric filaments (58). To monitor cytoskeletal changes, Mk progenitors subjected to Dyrk inhibition were assessed for F-actin content by staining with Phalloidin 594 and fluorescence microscopy. In primary human progenitors, as well as in the imMKCL line, both inhibitors strongly promoted actin incorporation into cortical filaments (Figure 6, A and B and Supplemental Figure 9A). A proteomic screen for DYRK1A substrates previously implicated F-actin stabilizers in the Ablim family as potential downstream targets (33). Within this family, Ablim2 demonstrated Mk-specific expression in human marrow samples and showed a cortical subcellular distribution pattern (ProteinAtlas database, ref. 59). Treatment of neonatal progenitors with Dyrk1 inhibitors markedly augmented the levels of both Ablim2 and its cofactor striated muscle activator of Rho signaling (STARS) (Figure 6, C and D, and Supplemental Figure 9, B and C; ref. 60), with Ablim2 displaying a cortical distribution similar to that of F-actin (Supplemental Figure 9B). A connection between DYRK1A and Ablim2 was further supported by retroviral transduction of adult progenitors in which enforcement of WT DYRK1A repressed Ablim2 levels while the dominant negative K188R enhanced its expression (Supplemental Figure 9D).

### Ablim2 resides upstream of actin and MKL1 in morphogenesis signaling.

Because it is controlled by Dyrk1 activity and its role in F-actin stabilization, Ablim2 was assessed for its participation in the morphogenetic effects of Dyrk inhibition. Neonatal CD34^+^ progenitors underwent lentiviral shRNA-mediated knockdowns using a standard approach in our lab (26). Two distinct hairpins each provided more than 80% knockdown (Supplemental Figure 10A), and both strongly suppressed polyploidization induced by Dyrk inhibitors (Figure 7, A–C). Ablim2 deficiency also blocked Filamin A induction as well as cytologic changes (Supplemental Figure 10, B and C). To determine the position of Ablim2 within the morphogenesis signaling pathway, we examined its influence on MKL1 and actin. Notably, knockdown of Ablim2 prevented both MKL1 nuclear translocation and F-actin formation in response to Dyrk inhibition (Figure 7, D–I), indicating its likely role as a critical upstream component in this pathway.

## Discussion

The changes in Mks that occur with ontogenic development most likely serve stage-specific physiologic needs, such as rapid expandability in early embryogenesis, a capacity for vascular and immune modulation during later embryonic development, and prevention of a hypercoagulable state in the maturing fetus (1, 5). However, infantile Mks, including those in neonates, are poorly suited for adult needs due to their limited capacity for platelet production. Defining pathways that control the ontogenic phenotype offers potential benefits in a variety of clinical scenarios, including thrombocytopenias in newborns and CB transplant recipients, as well as in optimizing systems to manufacture donor-independent platelets. In the last scenario, a capacity for rapid and efficient induction of adult-type morphogenesis in infantile Mks could eliminate a major bottle-neck in ex vivo production of clinical grade platelets, i.e., inefficiency of scale-up.

As a critical node in the natural transition from human fetal to adult megakaryopoiesis (26), the transcriptional coactivator MKL1 represents a rational target for ontogenic manipulation. Its signaling properties also make MKL1 a compelling target for enhancement of morphogenesis. Firstly, its activation may be amplified through feed-forward loops associated with cytoskeletal reconfiguration. Its transcriptional partner SRF programs focal adhesion and F-actin assembly (61), which then further enhances its own nuclear translocation and coactivation function. Its direct targets, Filamin A and Hic-5, which are deficient in fetal Mks (26) and induced by Dyrk inhibition (Figure 5, A and B), both augment MKL1 activity. Filamin A directly binds MKL1 to potentiate its response to F-actin formation (62) and also contributes to Mk polyploidization (63). Hic-5 exerts feed-forward activity by promoting F-actin stress-fiber maturation (51). Secondly, MKL1-SRF signaling may participate in lineage consolidation by limiting the potential of progenitors to adopt alternative differentiation pathways (64), potentially acting to suppress the leaky erythroid gene expression characteristic of fetal Mk (26, 65) (see also Supplemental Figure 1D). Recent single-cell RNA-Seq studies of human Mk from yolk sac, fetal liver, and adult marrow have highlighted heterogeneity at all stages, identifying subpopulations dedicated to either immune, hemostatic, or niche functions (66, 67). A comparison of yolk sac Mk with the later-stage fetal liver Mk identifies emergence in the latter of an MK4 subset that bears signatures of cell-substrate adhesion, TGFβ signaling, and induction of cytoskeletal factors including actin, MYL9, and vimentin (66). All of these signatures are associated with MKL1 activity (28), suggesting the emergence of an incipient morphogenesis program during the transition from yolk sac to fetal liver.

Endogenous stimuli known to control MKL1 activity include receptor-mediated signal transduction, the mechanical milieu, and nuclear lamina properties (28). Receptor engagement by the megakaryopoietic cytokine TPO induces rapid and transient MKL1 nuclear translocation in primary murine megakaryocytic progenitors in suspension culture (30). Environmental matrix stiffness provides a mechanical stimulus that promotes Mk morphogenesis via myosin heavy chain 9 signaling to MKL1 (21). Other mechanical stimuli that enhance Mk morphogenesis, such as shear stress and turbulence (20, 22), most likely also exert effects through MKL1 activation. Nuclear lamina components lamin A/C and emerin dictate nuclear compliance, which, in turn, affects actin network dynamics, MKL1 distribution (68), and megakaryocytic developmental potential (69). The clinical syndrome of Hutchinson-Gilford progeria, caused by mutant lamin A and enhanced nuclear envelope stiffness, includes significantly elevated platelet counts (70). Transgenic mouse studies directly demonstrate aberrant MKL1 activation by the progerin mutant of lamin A (71). Thus, several factors have been found to enhance Mk morphogenesis via MKL1 activation, further validating its importance as a target. However, these pathways lack sufficient potency for efficient ontogenic reprogramming and are not easily amenable to therapeutic manipulation.

The pathway identified herein enables control of an ontogenic switch by applying what we believe to be a novel strategy that targets molecular defects underlying infantile Mk morphogenesis. We previously identified IGF2BP3 blockade of MKL1 induction as a key mechanism restraining morphogenesis (26). The current study establishes an approach by which to bypass this blockade through reinforcement of MKL1 at the functional level (Figure 7J). Earlier studies have identified negative control of MKL1 exerted by multiple kinases. ERK-mediated phosphorylation of MKL1 on serine 454, located within a highly conserved central region, strongly enhances its interaction with G-actin and nuclear export (32). GSK3β-mediated phosphorylation on conserved serine 467 of the MKL1 paralog Myocardin, corresponding to serine 458 in MKL1, interferes with transcriptional activation function (34). Additional MKL1 phospho-sites have been mapped by Panayiotou, some with negative and others with positive effects (36). This group also identified an inhibitory effect of classical protein kinase C (cPKC) on F-actin formation, acting to block MKL1 nuclear translocation (36). Schneider et al. identified a similar inhibitory effect of DYRK1A on F-actin and MKL1, exerted by phosphorylation of Ablim proteins (33). In the current work, our screen ruled out participation of ERK, GSK3b and cPKC in the restraint of MKL1 function in infantile Mks (Supplemental Figure 1, A and B). Rather, our studies identified DYRK1A as the critical morphogenetic brake in megakaryopoiesis and showed that its activity can be manipulated to optimize platelet production. Availability of United States Food and Drug Administration-approved (USFDA-approved ) DYRK1A inhibitors will enable clinical trials in the near future. Toxicities observed with shRNA knockdown and biallelic knockout do raise the possibility that in vivo treatment with such inhibitors may have a narrow therapeutic window. However, ex vivo treatment strategies will likely allow greater flexibility. From a broader perspective, our results show that defining basic mechanisms governing fetal-to-adult transitions can yield targeted approaches toward the treatment of common clinical problems such as thrombocytopenia.

## Methods

### Cell culture.

Cryopreserved neonatal CB and adult PB CD34^+^ primary human progenitors were purchased, thawed, prestimulated, and subjected to unilineage Mk culture as previously described (26). Specifically, undifferentiated cells were expanded for 72 hours in prestimulation medium containing Iscove modified Dulbecco medium with 20% BITS 9500 (StemCell Technologies), 2 mM l-glutamine, 100 ng/ml stem cell factor (SCF; PeproTech), 100 ng/ml FLT3-ligand (PeproTech), 100 ng/ml TPO (PeproTech), and 20 ng/ml IL-3 (PeproTech). Unilineage Mk cultures contained the same basal medium, but with cytokines consisting of 40 ng/ml TPO, 25 ng/ml SCF, 100 ng/ml stromal-derived factor-α, and used fibronectin-coated wells. Cytokines were purchased from PeproTech. For prolonged expansion of undifferentiated human multipotent progenitors, CB CD34^+^ cells underwent 8 days of culture in prestimulation medium supplemented with 1 μM StemRegenin 1 (SR1, Selleckchem). Human iPSC–derived Mk progenitors were generated in-house as described previously (16). The cryopreserved iPSC-derived Mk progenitors were thawed and grown in serum-free differentiation (SDF) medium consisting of 75% IMDM with 25% Ham’s F12 medium, 0.5X N-2 supplement (Thermo Fisher Scientific), 1X B-27 supplement minus vitamin A (Thermo Fisher Scientific), 0.05% bovine serum albumin (Sigma-Aldrich), 50 μg/ml ascorbic acid (Sigma-Aldrich), 450 μM 1-thioglycerol (Sigma-Aldrich), 100 ng/ml human TPO (PeproTech), and 25 ng/ml human SCF (PeproTech). The iPSC-derived Mk cell line imMKCL TkDN-SEV2 clone 7, generated in-house was derived, cryopreserved, thawed, and expanded in suspension culture with doxycycline as previously published (43). For differentiation, cells underwent transfer to a recently optimized doxycycline-free induction medium, as described (22). Inhibitors of the MKL1-regulatory kinases cPKC (Go6983), MEK (U0126), and GSK3 (TDZD8) were purchased from Cayman Chemical, dissolved in DMSO and diluted in culture medium to final concentrations. The other commercially available inhibitors harmine (Dyrk family), INDY (DYRK1A/B), harmane (monoamine oxidase [MAO]), and harmaline (MAO) were purchased from Sigma-Aldrich, similarly dissolved in DMSO, and diluted. The highly selective DYRK1A inhibitors, EHT 1610 and FC 162, were synthesized as described previously (40, 41). These compounds were also dissolved in DMSO prior to use at the indicated concentrations. For ex vivo cultures of murine progenitors, marrow from mice treated with 5-fluorouracil underwent erythrocyte depletion with Gibco ACK lysing buffer (Thermo Fisher Scientific) followed by a cold PBS wash. To expand progenitors, cells were cultured 1 day in RPMI-1640 with 10% FBS, supplemented with murine cytokines from Peprotech (50 ng/ml SCF, 50 ng/ml IL-6, 20 ng/ml IL-3), as well as 50 μM 2-mercaptoethanol, 100 U/ml penicillin, and 100 U/ml streptomycin. For Mk differentiation cytokine composition was changed to 40 ng/ml human TPO plus 25 ng/ml murine SCF, and cultures continued for an additional 3 days.

### Cell transduction and transfection.

Retroviral expression constructs for WT and dominant negative DYRK1A (MIGR1-WtDyrk1a and MIGR1-Dyrk1aK188R) were provided by John D. Crispino (Northwestern University Feinberg School of Medicine, Chicago, Illinois, USA.) (31). Production of retroviral supernatants by transient transfection of these plasmids and matched parent vector into packaging lines, followed by progenitor transduction using repeated spinoculation, occurred as previously described (26, 72). For flow cytometric assessment of morphogenesis and for immunofluorescence, transduced cells were cultured an additional 5 days in megakaryocytic medium. For platelet release assays, transduced cells were cultured 11 days in megakaryocytic medium. Lentiviral constructs for shRNA-mediated knockdowns of *ABLIM2* consist of the pLKO.1 vectors produced by the RNAi Consortium (TRC) and purchased from Horizon Discovery (clone IDs.TRCN0000146452 (A8) and TRCN0000146821 (A9). Production of lentiviral supernatants by transient cotransfection of pLKO.1 vectors with packaging plasmids into 293T cells, purchased from the ATCC, followed by spinoculation of preexpanded progenitors took place as per descriptions (26, 29). Cells after spinoculation underwent selection in prestimulation medium with 1.2 μg/ml puromycin for 3 days followed by differentiation for 5 days in Mk medium with 0.5 μg/ml puromycin.

### Mice.

*Mkl1^–/–^* mice generated as described previously (73) were backcrossed onto C57BL/6 and provided by Diane Krause (Yale School of Medicine, New Haven, Connecticut, USA.). *Dyrk1a^fl/fl^* mice on a C57BL/6 background (C57BL/6-*Dyrk1a^tm1Jdc^*/J) were purchased from The Jackson Laboratory. The megakaryocytic deleter strain *Pf4^Cre^* on a C57BL/6 background (C57BL/6-Tg(Pf4-icre)Q3Rsko/J) was purchased from The Jackson Laboratory. Immunodeficient xenotransplant recipients consisted of NOD/SCID IL-2Rγ-null mice (NOD.Cg-*Prkdc^scid^ Il2rg^tm1Wjl^*/SzJ, referred to as NSG), purchased from The Jackson Laboratory. PCR genotyping of genomic DNA is described in Supplemental Methods. Procedures included injection of a single i.p. dose of 5-fluorouracil (Teva Pharmaceutical) at 75 mg/kg for 7 days before marrow harvest for ex vivo Mk cultures. Transplantation of human CB Mk into NSG mice followed our recently described approach (15), as did the quantitation of human circulating platelets and human Mks trapped in the lung. For infusion of platelets, the mice each received 8.5 × 10^6^ platelets harvested from Mk cultures of CB CD34^+^ cells on day 11; as a positive control, a separate cohort of mice were infused with the same number of normal donor platelets.

### Flow cytometry analysis.

Human and murine progenitors cultured in Mk medium were washed, stained for surface markers with and without DNA content, and analyzed as recently published (26). For analysis of primary bone marrow Mk, murine bone marrow was harvested, processed, and labeled as described previously (26). For all flow studies, assessments of ploidy, size (FSC), and granularity (SSC) were conducted on gated viable, singlet, CD41^+^ Mk; in retroviral transduction experiments the gating additionally included GFP^+^ cells, and BV421, rather than FITC, was the fluorochrome for staining CD41. For studies prior to June 2017, analytical software consisted of FlowJo (TreeStar); later studies employed FCS Express 6 (De Novo). For the in vitro platelet release assay, culture-derived platelets underwent isolation and flow cytometric quantitation as described (15, 26). For platelet activation assays, culture-derived platelets were treated with 40 μM thrombin receptor activating peptide 6 (TRAP-6) (Thermo Fisher Scientific), plus 100 μM adenosine 50-diphosphate (ADP) (Sigma-Aldrich). Samples were stained with anti-human CD61 and CD62P, or the corresponding isotype controls (Becton Dickinson), and subjected to flow cytometry (15). Donor-derived platelets were stimulated and labeled identically and utilized as controls.

### Microscopy.

For cytology by light microscopy, cells underwent cytospin at a density of 2.5 × 10^4^ cells per glass slide followed by Wright stain (Sigma-Aldrich). For light microscopic histology, formalin-fixed, decalcified, paraffin-embedded mouse femora were sectioned at 4 μm, deparaffinized with EZ Prep (Ventana), and subjected to H&E staining. For IF, cells underwent cytospin at a density of 4 × 10^4^ cells per glass slide, followed by fixation 10 minutes in 4% paraformaldehyde/PBS at room temperature. Cells were washed twice with PBS, and permeabilized/blocked for 1 hour in blocking buffer (PBS with 2% FBS, 2% BSA, and 0.1% Triton X-100). Primary antibodies consisted of rabbit polyclonal anti-MKL1 (Bethyl Laboratories, A302-201A) at 1:100, or rabbit polyclonal anti-Ablim2 (Sigma-Aldrich, HPA035808) at 1:100 in blocking buffer applied overnight at 4°C. After 4 washes with blocking buffer, the secondary incubation consisted of blocking buffer with goat anti-rabbit Alexa Fluor 488 (Thermo Fisher Scientific, A-11008) at 1:300, DAPI, and (where indicated) Alexa Fluor 594 Phalloidin (Invitrogen, A12381) at 1:20 for 60 minutes at room temperature. Slides were washed 4 times with blocking buffer and once with PBS and underwent coverslip mounting with Vectashield medium (Vector Laboratories, H-1000). Cytologic imaging by light microscopy employed an Olympus BX51 microscope equipped with an Olympus DP70 digital camera. Objective lenses consisted of Uplan Fl 20×/0.50 NA and Uplan Fl 40×/0.75 NA. Image acquisition and processing used Photoshop CS2/9.0 and CS3/10.0 (Adobe Systems) and Fiji (ImageJ V1, NIH). Histologic imaging by light microscopy employed a Hamamatsu NanoZoomer S360 scanner followed by processing with Fiji. For IF, single-plane mid-nucleus images were captured with a Zeiss LSM 700 confocal microscope using the 63× objective. Images were processed using Fiji. MKL1 nuclear signal was quantitated from the green signal that overlapped with DAPI. Cytoplasmic MKL1 was derived by subtracting green nuclear signal from the pan-cellular green signal. To quantitate F-actin, signals from staining with phalloidin Alexa 594 were divided by number of cells/field to yield cellular mean fluorescence intensity (MFI) using Fiji. For all IF experiments, at least 50 cells per experiment were used for quantitation. Transmission electron microscopy of culture-derived and normal donor platelets was conducted with a HitachiH-7650 transmission electron microscope (Hitachi High-Technologies Instrumentation) linked to a SIA (Scientific Instruments and Applications) digital camera (15).

### Cell extraction, fractionation and immunoblot.

Cultured imMKCL cells underwent washing with PBS, re-suspension in ice-cold Buffer A (10 mM HEPES-KOH pH 7.9, 1.5 mM MgCl_2_, 10 mM KCl, 0.5 mM DTT, 0.2 mM PMSF, 1× cOmplete EDTA-free protease inhibitor cocktail [Roche], 40 μM calpeptin [Sigma-Aldrich]), incubation for 10 minutes followed by vortexing for 10 seconds and pelleting with a 10 second microfuge pulse. The cell pellets were resuspended in ice-cold Buffer B (10 mM HEPES-KOH pH 7.5, 10 mM MgCl_2_, 10 mM KCl, 1 mM DTT, 1 mM EDTA, 1 x protease inhibitor cocktail, 40 μM calpeptin, 0.2% NP-40), incubated for 10 minutes, and microfuged for 5 minutes at 2,040*g*, with supernatants collected as “Cytoplasmic Fractions.” Pellets underwent washing with Buffer B and then re-suspension in ice cold Buffer C (20 mM HEPES-KOH pH 7.5, 1.5 mM MgCl_2_, 450 mM NaCl, 1 mM DTT, 0.5 mM EDTA, 1 × protease inhibitor cocktail, 40 μM calpeptin, 0.5% NP-40), incubation 10 minutes, microfuge 5 minutes at 8,160*g*, and collection of supernatants designated “Nuclear Fractions.” For immunoblotting, subcellular fractions were mixed with equal volumes of 2 × Laemmli sample buffer (60 mM Tris-HCl, pH 6.8, 2% SDS, 100 μM dithiothreitol, 10% glycerol, 0.01% bromophenol blue). For direct analysis of whole cell lysates, intact cell pellets underwent lysis in an equal volume of 2 × Laemmli supplemented with protease and phosphatase inhibitors (cOmplete and PhosSTOP, Roche) followed by shearing of DNA. All samples were boiled for 5 minutes, followed by SDS-PAGE and immunoblot as described (74). Primary antibodies and incubation conditions are provided in Supplemental Table 3. Densitometry data were acquired on a GS800 calibrated densitometer (Bio-Rad) and analyzed with Quantity One software (Bio-Rad; see complete unedited blots in the supplemental material).

### RNA-seq.

Adult and neonatal CD34^+^ cells cultured in megakaryocytic medium 4 days with DMSO or Dyrk inhibitors underwent removal of dead cells by Ficoll gradient centrifugation (GE Healthcare Bio-Sciences AB). Mks were then purified using anti-CD61 microbeads (Miltenyi Biotec) combined with magnetic sorting on an autoMACS Pro Separator (Miltenyi Biotec). RNA isolation employed the RNeasy Plus Mini Kit (Qiagen), followed by quality control using an Agilent 2100 (Agilent). The cDNA library was constructed using an NEBNext Ultra II RNA Library Prep Kit for Illumina (New England BioLabs), and sequencing was conducted on an NovaSeq 6000 Sequencing System (Illumina). The raw sequencing data underwent quality control analysis and filtering using the NG SQC Tool Kit version 2.3.3, followed by alignment of high-quality reads to the hg38 genome using HISAT version 2.1.0, read count determination with StringTie version 1.3.3b, and differential expression analysis with Ballgown version 2.6.0 (75). Gene set enrichment analysis was conducted using Enrichr (56). Sequence files have been deposited in GEO (GSE195671).

### Statistics.

Individual results shown are representative of at least 3 independent experiments. Graphs were generated using GraphPad Prism version 9.1.2 and depict mean values from at least 3 independently conducted experiments ± SEM. Single, pairwise comparisons employed 1-tailed Student’s *t* tests; multiple comparisons employed 1-way ANOVA with Tukey’s *post hoc* test using SPSS Statistics V26 (IBM). Gene set overlaps were analyzed using the Stat Trek Hypergeometric Calculator (https://stattrek.com/online-calculator/hypergeometric.aspx). A *P* value less than 0.05 was considered significant.

### Study approval.

CD34^+^ cells purchased from the Fred Hutchinson Cancer Research Center (Seattle, Washington, USA), AllCells (Alameda, California, USA), and StemCell Technologies (Vancouver, British Columbia, Canada; and Cambridge, Massachusetts, USA) were originally obtained from donors with informed consent and IRB approval at institutions of origin. Animal studies were approved and performed in compliance with the University of Virginia and Icahn School of Medicine at Mount Sinai IACUC committees and guidelines.

## Author contributions

KEE performed experiments, conceptualized and interpreted data, and wrote the manuscript. AB performed cell culture experiments and assisted with mouse studies. GM and CMC conducted NSG xenotransplantation and in vitro platelet release assays. LLD assisted with mouse breeding and analysis. RKS assisted with flow cytometry studies. APB, CF, and TB synthesized selective Dyrk1 inhibitors. SWM generated the *Mkl1*^–/–^ mouse strain. KE developed the iPSC-derived imMKCL line. CJ, DLF, and PG developed the iPSC-derived Mks. CIR supervised NSG xenotransplantation and in vitro platelet release experiments and interpreted data. SS, XS, FQ, and RC processed and analyzed RNA-Seq data under the supervision of HL. ANG conceived of and supervised the project, interpreted the data, and wrote the manuscript.

## Supplementary Material

Supplemental data

## Figures and Tables

**Table 1:**
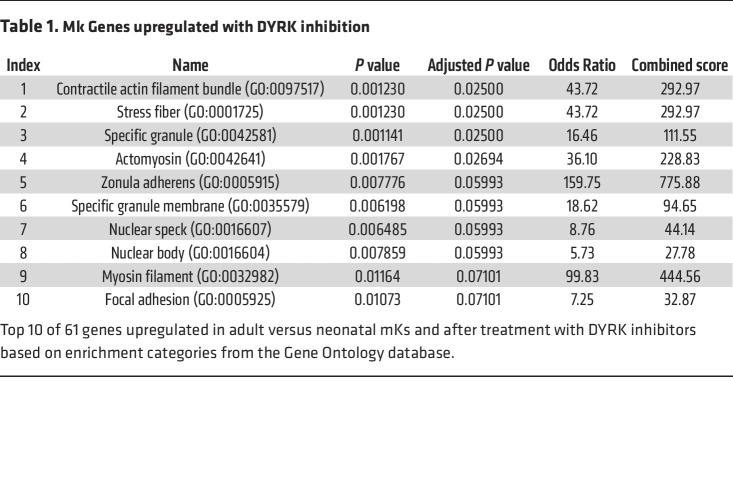
Mk Genes upregulated with DYRK inhibition

## Version 1 08/04/2022

In-Press Preview

## Version 2 10/03/2022

Electronic publication
