# Supplementary material for: Relieving DYRK1A repression of MKL1 confers an adult-like phenotype to human infantile megakaryocytes
Source: J Clin Invest. 2022 Oct 3;132(19):e154839. doi: 10.1172/JCI154839 (PMC9525118; doi:10.1172/JCI154839)
Supplement: Supplemental data [file jci-132-154839-s029.pdf]

Supplemental Figures

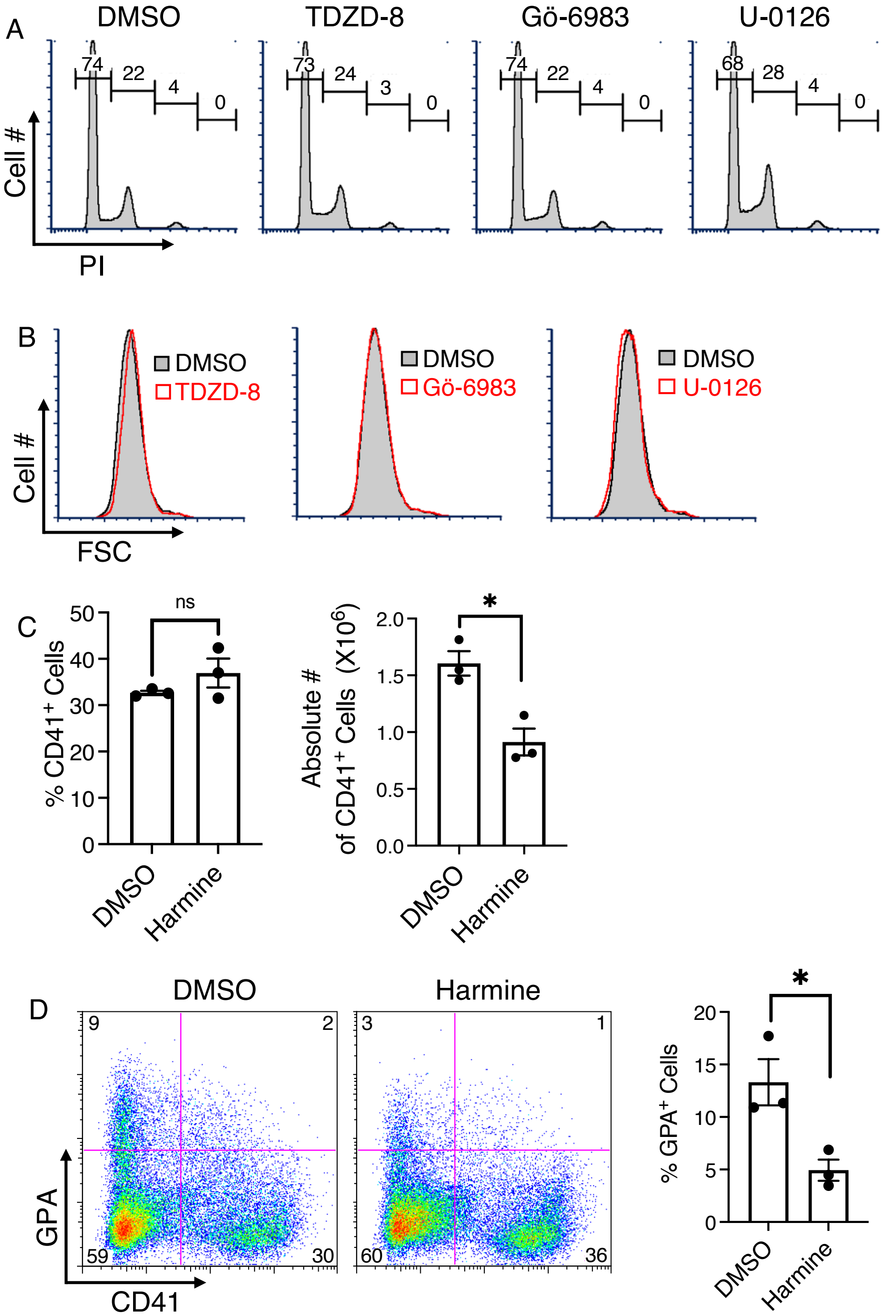

Supplemental Figure 1. Induction of adult-type morphogenesis in neonatal megakaryocytes (Mk) treated with Dyrk kinase inhibitors. (**A-B**) Cord blood $CD34^+$ cells were cultured 6 days in Mk medium ± either 5 μM GSK3 inhibitor TDZD-8, 1 μM PKC inhibitor Gö-6983 or 5 μM MEK inhibitor U-0126 followed by flow cytometry after costaining with FITC-anti-CD41 and PI. (**A**) Mk polyploidization (PI). (**B**) Mk size (FSC). (**C**) Percentage (%) and numbers (#) of $CD41^+$ cells in day 6 Mk cultures of cord blood $CD34^+$ cells ± 5 μM harmine. Graphs: mean ± SEM for 3 independent experiments. *$P < 0.05$; ns: not significant, Student's *t* test. (**D**) Assessment of "leaky" erythroid differentiation. Cord blood $CD34^+$ cells cultured 6 days in Mk medium ± 5 μM harmine underwent flow cytometry analysis with PE-anti-GPA and APC-anti-CD41. Graph: mean % GPA^+ ± SEM for 3 independent experiments. *$P < 0.05$, Student's *t* test.

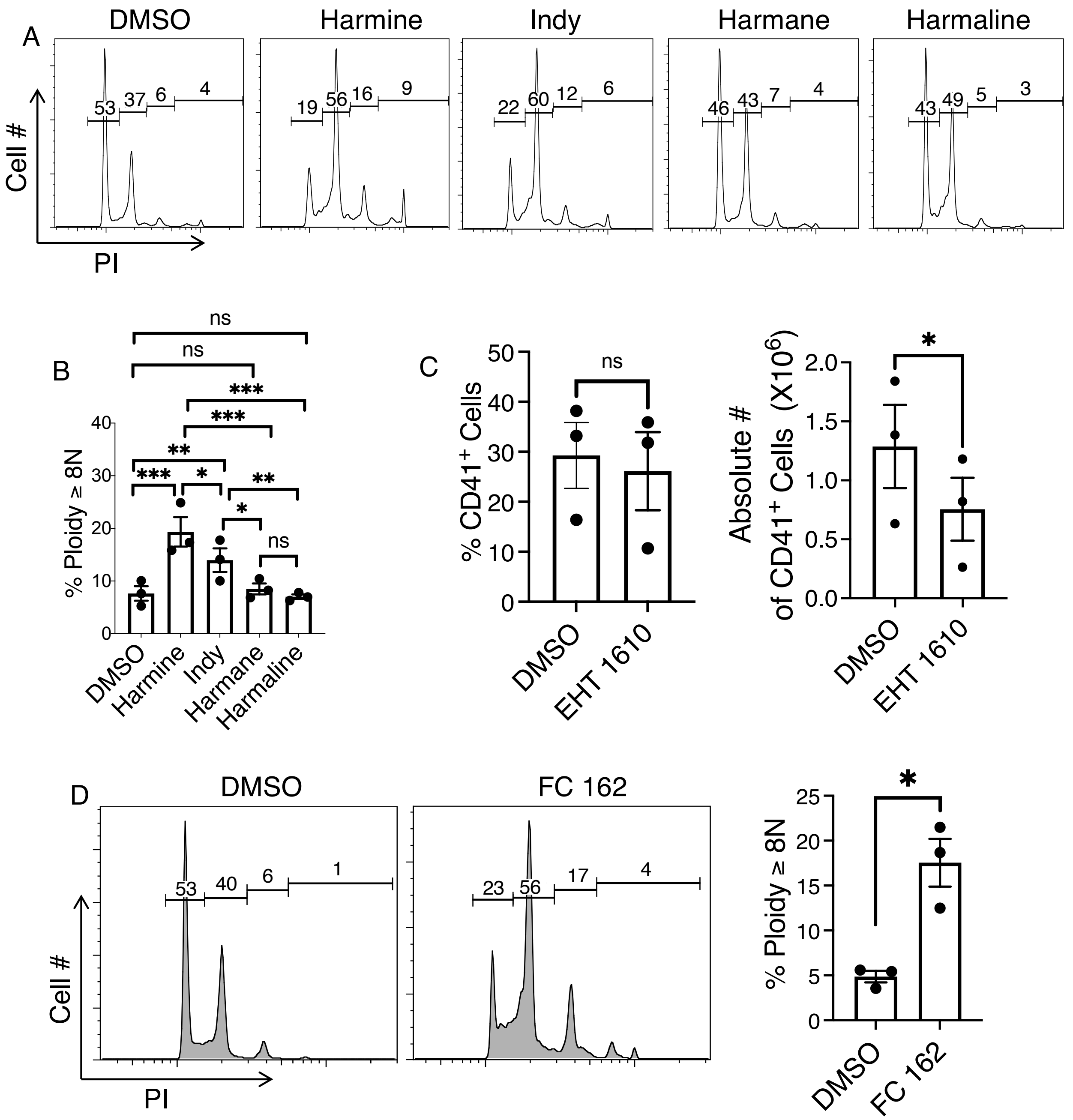

Supplemental Figure 2. Induction of adult-type morphogenesis in neonatal megakaryocytes (Mk) treated with Dyrk kinase inhibitors. (**A**) Dyrk inhibition enhanced morphogenesis of neonatal Mk morphogenesis. Cord blood $CD34^+$ cells cultured 6 days in Mk medium ± either 5 µM Dyrk1 inhibitors (Indy, harmine) or related monoamine oxidase inhibitors (harmane, harmaline) followed by flow cytometry after costaining with FITC-anti-CD41 and PI. (**B**) Graph: mean % Mk $\geq$ 8N ± SEM for 3 independent experiments. *P < 0.05, **P < 0.01, ***P < 0.005, ns: not significant, one-way ANOVA with Tukey's post hoc test. (**C**) Percentage and numbers of $CD41^+$ cells in day 6 Mk cultures of cord blood $CD34^+$ cells ± 5 µM EHT 1610. Graphs: mean ± SEM for 3 independent experiments. *P < 0.05; ns: not significant, Student's *t* test. (**D**) Flow cytometric analysis of cells as in (**A**) but treated with 5 µM selective Dyrk1a inhibitor FC 162. Graph: mean % Mk $\geq$ 8N ± SEM for 3 independent experiments. *P < 0.05, Student's *t* test.

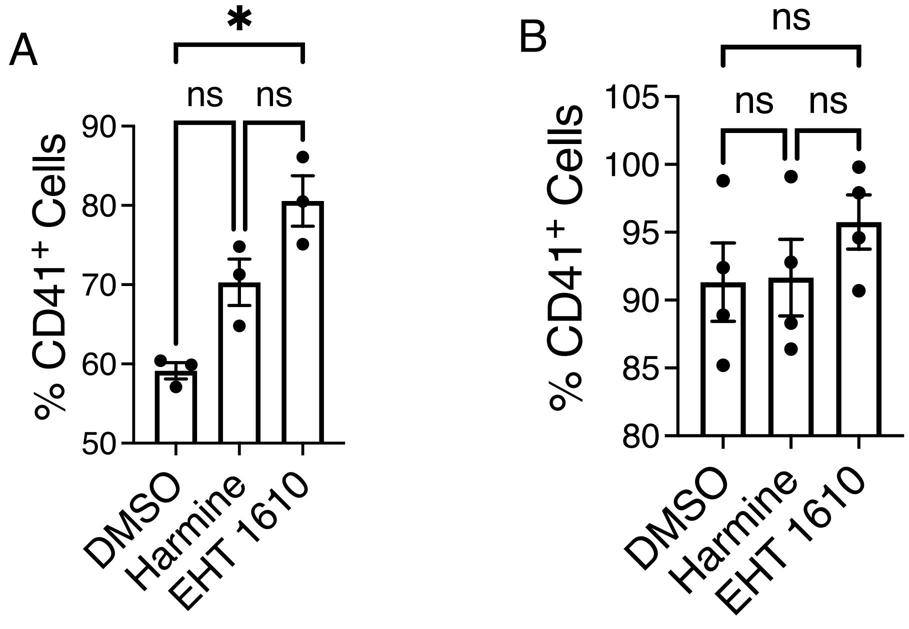

Supplemental Figure 3. Effect of Dyrk1 inhibitors on pluripotent stem cell-derived megakaryocytes (Mk). (**A**) Human iPSC-derived Mk progenitors cultured 5 days in serum-free differentiation medium ± 2.5 μM inhibitors (harmine or EHT 1610). Graph: mean % $CD41^+$ cells ± SEM for 3 independent experiments. **P* < 0.05; ns: not significant, one way ANOVA with Tukey's post hoc test. (**B**) Conditionally immortalized imMKCL cells cultured 6 days in doxycycline-free differentiation medium ± 5 μM inhibitors. Graph: mean % $CD41^+$ cells ± SEM for 4 independent experiments. ns: not significant, one way ANOVA with Tukey's post hoc test.

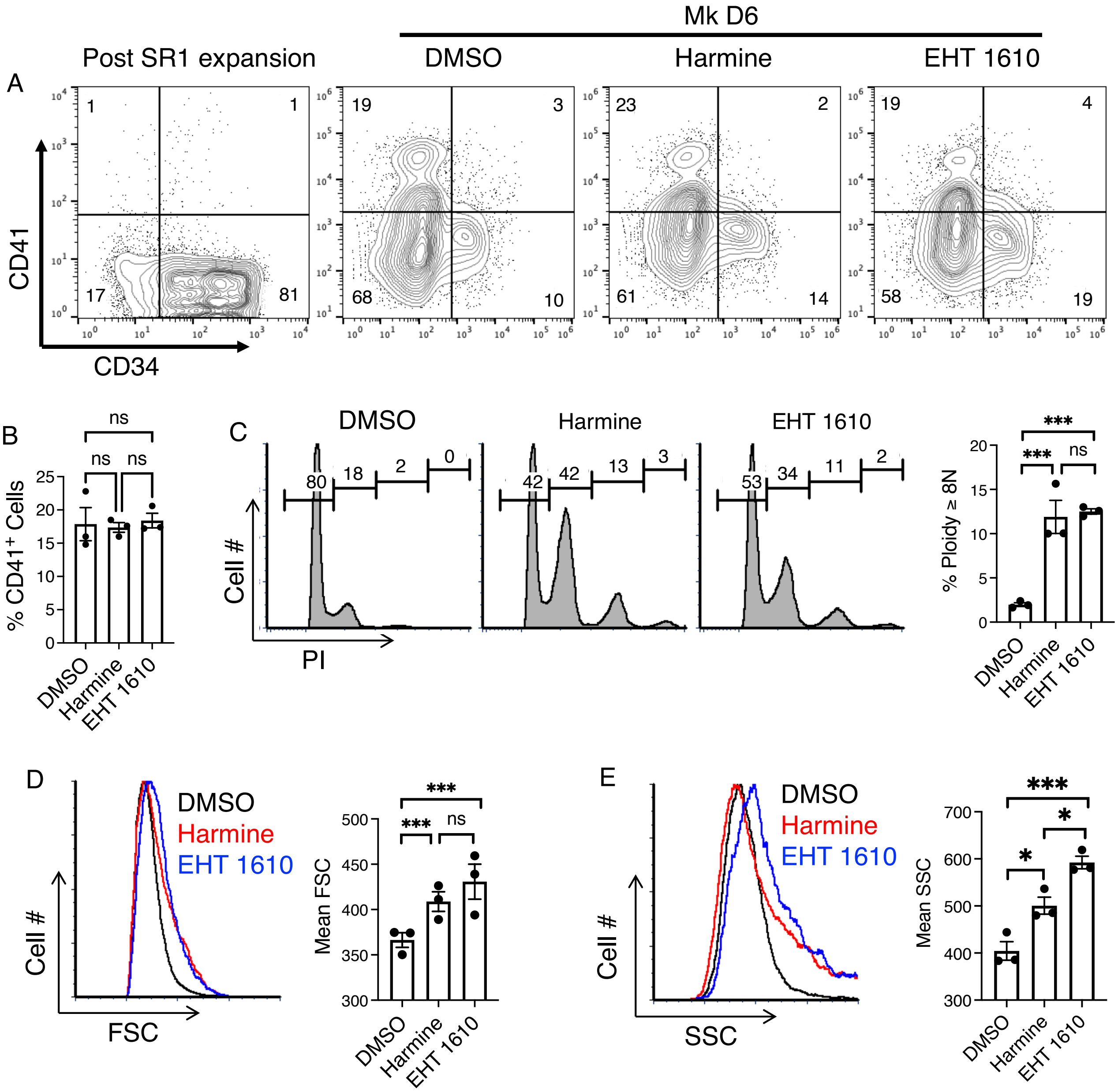

Supplemental Figure 4. Dyrk1 inhibitors elicit adult-type morphogenesis in neonatal megakaryocytes (Mk) derived from pre-expanded CD34+ progenitor cells. (**A**) Cord blood CD34+ cells underwent 8 days of expansion in pre-stimulation medium with 1 μM StemRegenin 1 (SR1) (left panel: Post SR1 expansion). The expanded cells were then cultured an additional 6 days in Mk medium ± 5 μM harmine or EHT 1610 (right three panels: Mk D6). Cells costained with PE-anti-CD34 and APC-anti-CD41 antibodies were analyzed by flow cytometry. (**B-E**) Cells from Mk D6 were costained with FITC-anti-CD41 and PI. (**B**) Percentage of CD41+ cells (Mk). (**C**) Mk polyploidization (PI). (**D**) Mk size (FSC). (**E**) Mk complexity/granulation (SSC). Graphs for **B-D**: mean ± SEM for 3 independent experiments. *$P < 0.05$; ***$P < 0.005$; ns: not significant, one way ANOVA with Tukey's post hoc test.

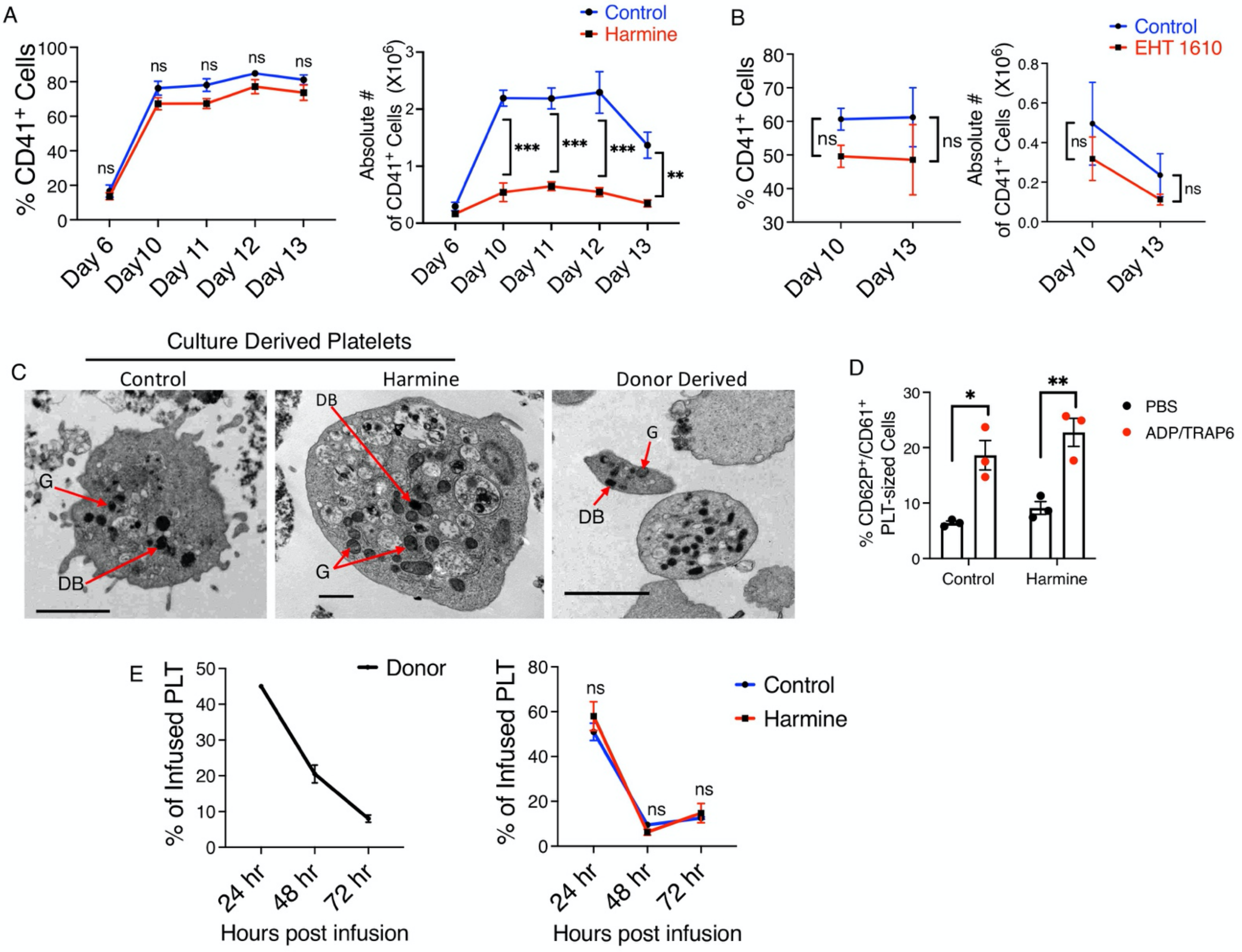

Supplemental Figure 5. Characterization of culture-derived platelets. **(A-B)** Percentage and number of CD41$^+$ cells used for platelet release assays. Cord blood CD34$^+$ cells were cultured up to 13 days in Mk medium ± 5 µM harmine or 2.5 µM EHT 1610. Graphs: means ± SEM for 4 independent experiments. **P < 0.01; ***P < 0.005; ns: not significant, one way ANOVA with Tukey's post hoc test. (**C**) Transmission electron microscopy (TEM) on culture- and donor-derived platelets showing dense bodies (DB) and alpha granules (G). Magnification: 6000x, scale bar 1 µm. (**D**) Platelet agonist treatment with ADP/TRAP6 followed by labelling with APC-anti-CD61 and FITC-anti-CD62P. Gating was based on size and CD61$^+$ characteristics of similarly treated normal donor platelets. Graph: mean percentage CD62P$^+$ platelets ± SEM for 3 independent experiments. *P < 0.05; **P < 0.01, one way ANOVA with Tukey's post hoc test. (**E**) *In vivo* life span of culture-derived platelets. 8.5 x 10^6 platelets from cord blood CD34$^+$ cells cultured 11 days Mk medium ± 2.5 µM harmine were infused into irradiated NSG mice (right). As a control, normal donor platelets were infused into a separate cohort of mice (left). Human platelets were quantified by flow cytometry (see Figure **3C**). Graphs: human platelets as percentage of initial infusion ± SEM, n = 4. ns: not significant, one way ANOVA with Tukey's post hoc test.

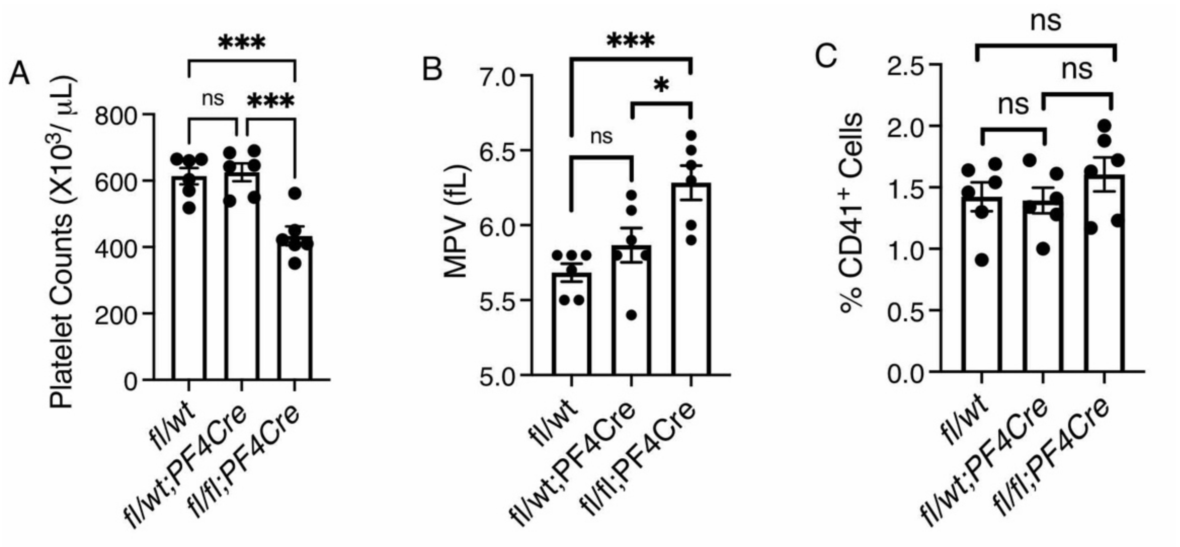

Supplemental Figure 6. Platelet counts, platelet size and megakaryocyte frequency in *Dyrk1a* conditional knockout mice. (**A-B**) Platelet counts and mean platelet volume (MPV) in peripheral blood samples from indicated strains. (**C**) Flow cytometry on marrow samples stained with FITC-anti-CD41. Graphs in **A-C**: means ± SEM, n = 6/group. **P* < 0.05; ****P* < 0.005; ns: not significant, one way ANOVA with Tukey's post hoc test.

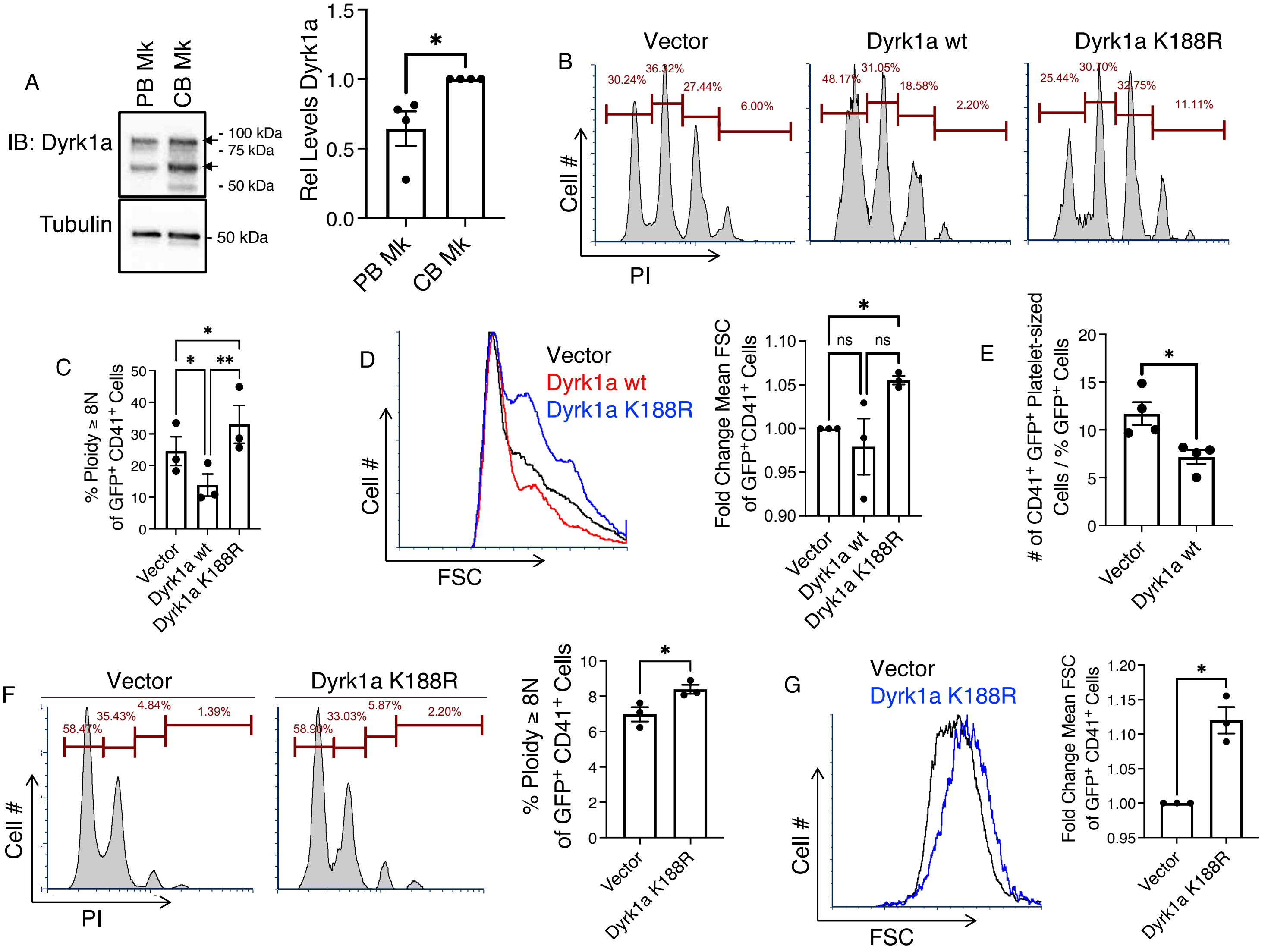

Supplemental Figure 7. Expression and function of Dyrk1a in primary adult and neonatal megakaryocytes (Mk). (**A**) Expression of Dyrk1a in adult and neonatal Mk. Immunoblot (IB) of whole cell lysates derived from adult peripheral blood (PB) or cord blood (CB) progenitors cultured 6 days in Mk medium. Arrows: Dyrk1a isoforms. Graph: mean relative Dyrk1a levels normalized to tubulin ± SEM for 4 independent experiments. *$P < 0.05$, Student's *t* test. (**B-E**) Adult $CD34^{+}$ cells transduced with retroviral expression constructs and cultured 5 days in Mk medium were stained with BV421-anti-CD41 antibody and propidium iodide (PI), followed by flow cytometry with gating on viable, singlet, transduced (GFP^{+}), $CD41^{+}$ cells. For platelet release assay, transduced cells were cultured 11 days, and analysis, conducted as in Figure 3A-B, additionally gated on GFP^{+} platelets. (**B-C**) Adult Mk polyploidization (PI). (**D**) Adult Mk Size (FSC). (**E**) Adult platelet release normalized for progenitor transduction efficiency. Graphs for **C-D**: mean ± SEM for 3 independent experiments. *$P < 0.05$; **$P < 0.01$; ns: not significant, one way ANOVA with Tukey's post hoc test. Graph for **E**: mean ± SEM for 4 independent experiments. *$P < 0.05$, Student's *t* test. (**F-G**) Neonatal $CD34^{+}$ cells transduced with retroviral expression constructs and cultured 5 days in Mk medium were stained with BV421-anti-CD41 antibody and propidium iodide (PI), followed by flow cytometry with gating on viable, singlet, transduced (GFP^{+}), $CD41^{+}$ cells. (**F**) Neonatal Mk polyploidization (PI). (**G**) Neonatal Mk Size (FSC). Graphs for **F-G**: mean ± SEM for 3 independent experiments. *$P < 0.05$, Student's *t* test.

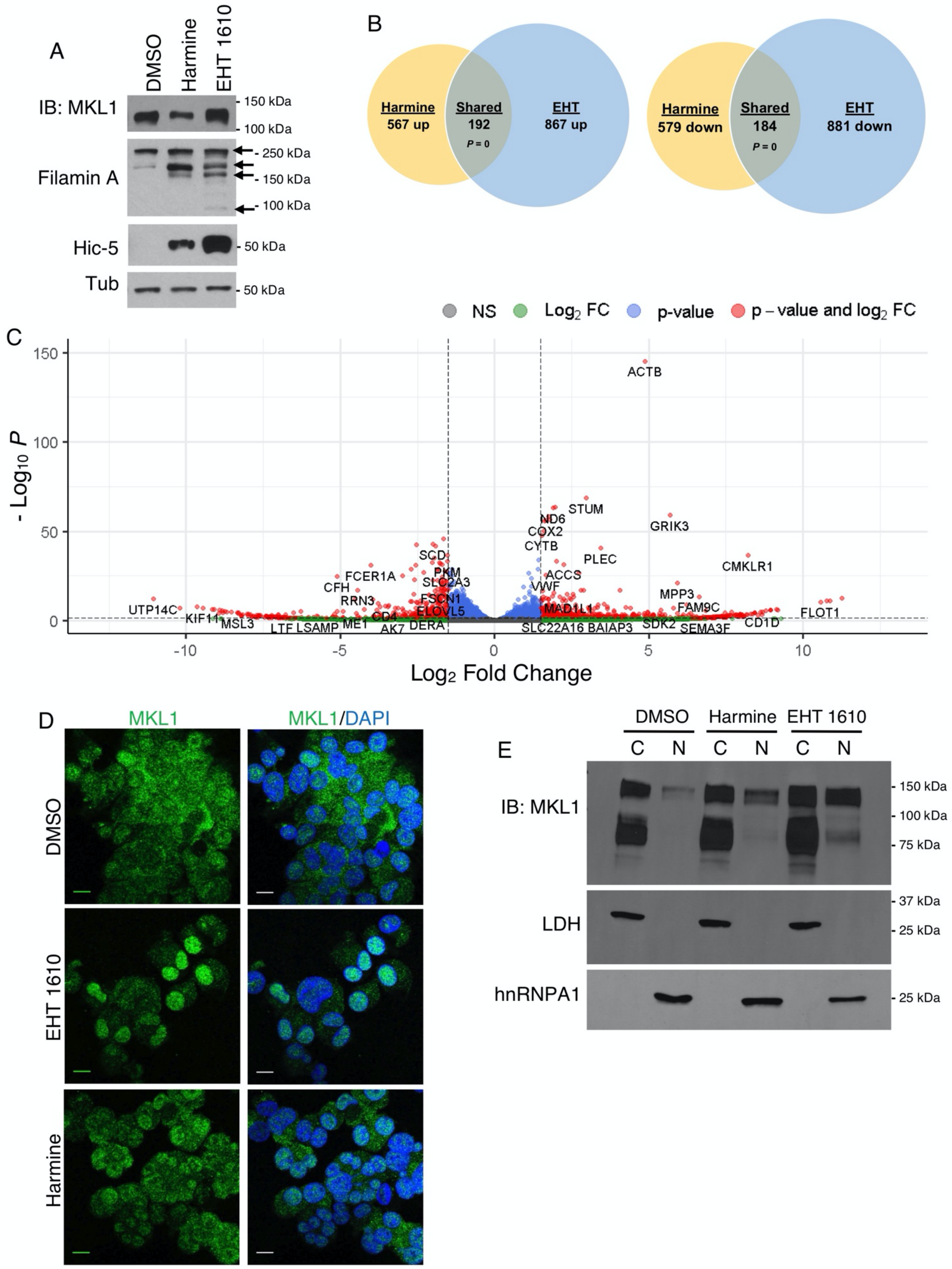
A
DMSO
Harmine
EHT 1610
IB: MKL1
Filamin A
Hic-5
Tub
150 kDa
100 kDa
250 kDa
150 kDa
100 kDa
50 kDa
50 kDa
B
Harmine
567 up
Shared
192
P = 0
EHT
867 up
Harmine
579 down
Shared
184
P = 0
EHT
881 down
C
NS
Log2 FC
p-value
p – value and log2 FC
- Log10 P
Log2 Fold Change
ACTB
STUM
ND6
COX2
CYTB
GRIK3
PLEC
CMKLR1
ACCS
VWF
MPP3
FAM9C
MAD1L1
FLOT1
CD1D
SDK2
SEMA3F
SLC22A16 BAIAP3
SCD
PKM
SLC2A3
FSCN1
ELOVL5
FCER1A
CFH
RRN3
CD4
DERA
AK7
ME1
LTF LSAMP
MSL3
KIF11
UTP14C
D
MKL1
MKL1/DAPI
DMSO
EHT 1610
Harmine
E
DMSO
Harmine
EHT 1610
C
N
IB: MKL1
LDH
hnRNPA1
150 kDa
100 kDa
75 kDa
37 kDa
25 kDa
25 kDa

Supplemental Figure 8. MKL1 regulation by Dyrk1 inhibitors. (**A**) Expression of MKL1 and its targets. imMKCL cultured 6 days in doxycycline-free induction medium ± 5 μM inhibitors underwent IB of whole cell lysates. Results shown are representative of 2 independent experiments. Arrows: Filamin A isoforms. (**B**) Transcriptomic effects in human megakaryocyte precursors of two different Dyrk inhibitors. Cord blood $CD34^+$ cells cultured 4 days in Mk medium ± 5 μM inhibitors underwent purification of $CD61^+$ cells followed by RNA-seq. Shown are overlapping genes (Shared) with hypergeometric *P* values; n = 3 independent experiments. (**C**) Transcriptomic effects of Dyrk inhibition in later-stage neonatal megakaryocytes. Cord blood $CD34^+$ cells cultured 11 days in Mk medium ± 2.5 μM harmine underwent RNA-seq. Volcano plot represented as $\log_2$ fold change versus $-\log_{10}$ *P* value for two independent experiments. (**D**) MKL1 localization. imMKCL cultured 2 days as in (**A**) underwent IF staining then confocal microscopy (Zeiss LSM700, original magnification x 630, scale bar 10 μm). Results shown are representative of 2 independent experiments. (**E**) Biochemical fractionation of imMKCL using differential salt extraction. Cells cultured as in (**D**) underwent IB of indicated fractions (C: cytoplasmic; N: nuclear). Results shown are representative of 3 independent experiments.

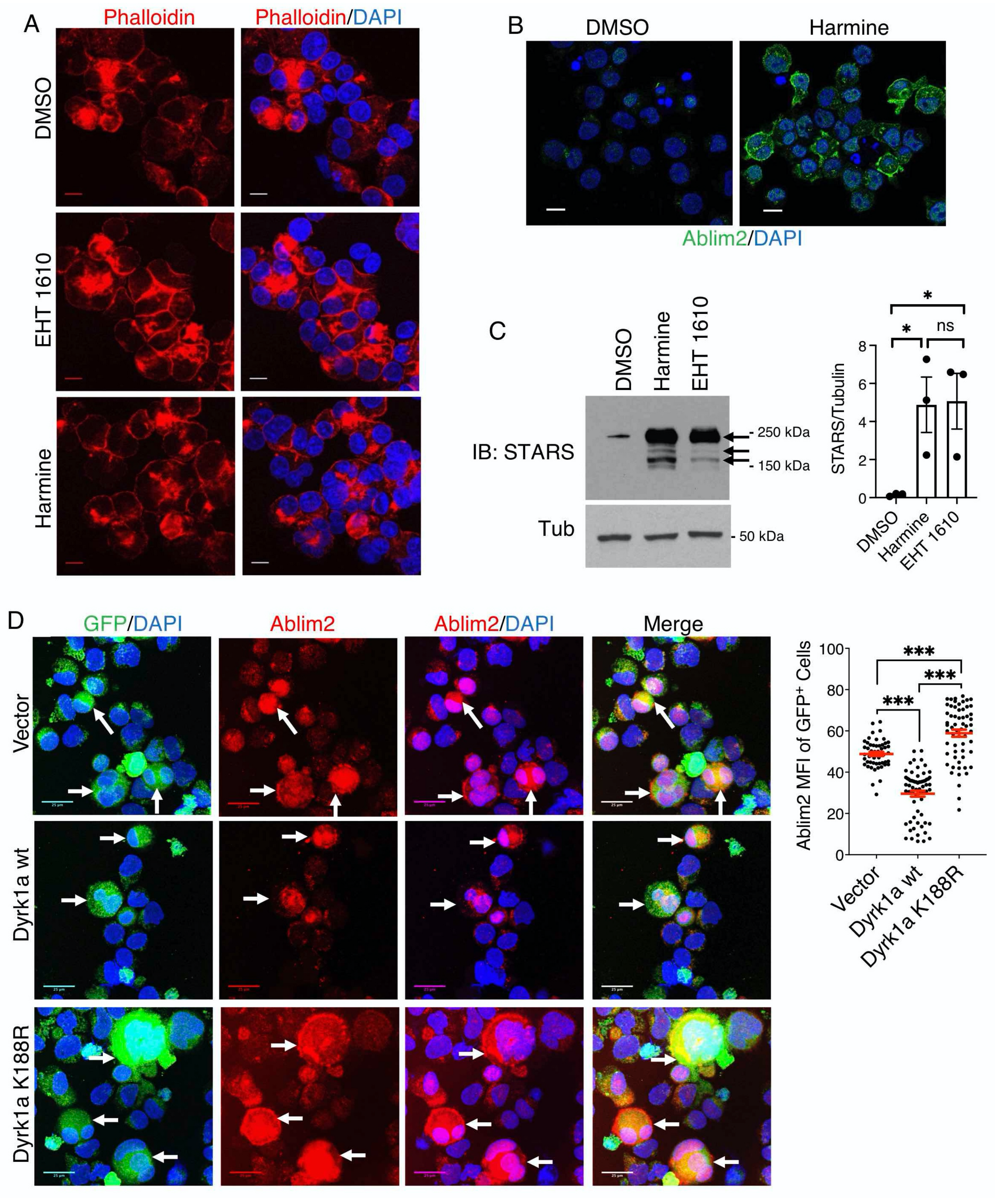
A
Phalloidin
Phalloidin/DAPI
DMSO
EHT 1610
Harmine
B
DMSO
Harmine
Ablim2/DAPI
C
DMSO
Harmine
EHT 1610
IB: STARS
Tub
- 250 kDa
- 150 kDa
- 50 kDa
STARS/Tubulin
*
*
ns
D
GFP/DAPI
Ablim2
Ablim2/DAPI
Merge
Vector
Dyrk1a wt
Dyrk1a K188R
Ablim2 MFI of GFP⁺ Cells
***
***
***

Supplemental Figure 9. Changes in actin cytoskeleton and actin-associated factors in association with Dyrk1 inhibition. (**A**) Induction of F-Actin. imMKCL cultured 2 days in doxycycline-free induction medium ± 5 μM inhibitors were stained with indicated dyes followed by confocal microscopy (Zeiss LSM700, original magnification x 630, scale bar 10 μm). Results shown are representative of 2 independent experiments. (**B**) Induction of Ablim2. Neonatal $CD34^+$ progenitors cultured 5 days in megakaryocyte medium ± 5 μM harmine underwent IF staining for Ablim2 (Green) followed by confocal microscopy (Zeiss LSM700, original magnification x 630, scale bar 10 μm). Results shown are representative of 3 independent experiments. (**C**) Induction of STARS. Cells as in (**B**) ± 5 μM inhibitors underwent IB of whole cell lysates. Graph: tubulin-normalized densitometry signals ± SEM for 3 independent experiments. **P* < 0.05; ns: not significant, one way ANOVA with Tukey's post hoc test. Note that the tubulin loading control image is the same as used in Figure 5A. (**D**) Effect of enforced Dyrk1a wild type (wt) or dominant negative mutant (K188R) on Ablim2 expression. Adult $CD34^+$ cells transduced with the indicated retroviral constructs were cultured 5 days in Mk medium. The cells underwent immunofluorescent staining for Ablim2 (Alexa-594 secondary antibody) followed by confocal microscopy (Zeiss LSM700, original magnification x 630, Z-stack image, scale bar 25 μm). White arrows: GFP^+ cells. Graph: Alexa-594 (Ablim2 signal) mean fluorescent intensity (MFI) in $\geq$ 50 GFP^+ cells ± SEM for two independent experiments. ****P* < 0.005, one way ANOVA with Tukey's post hoc test.

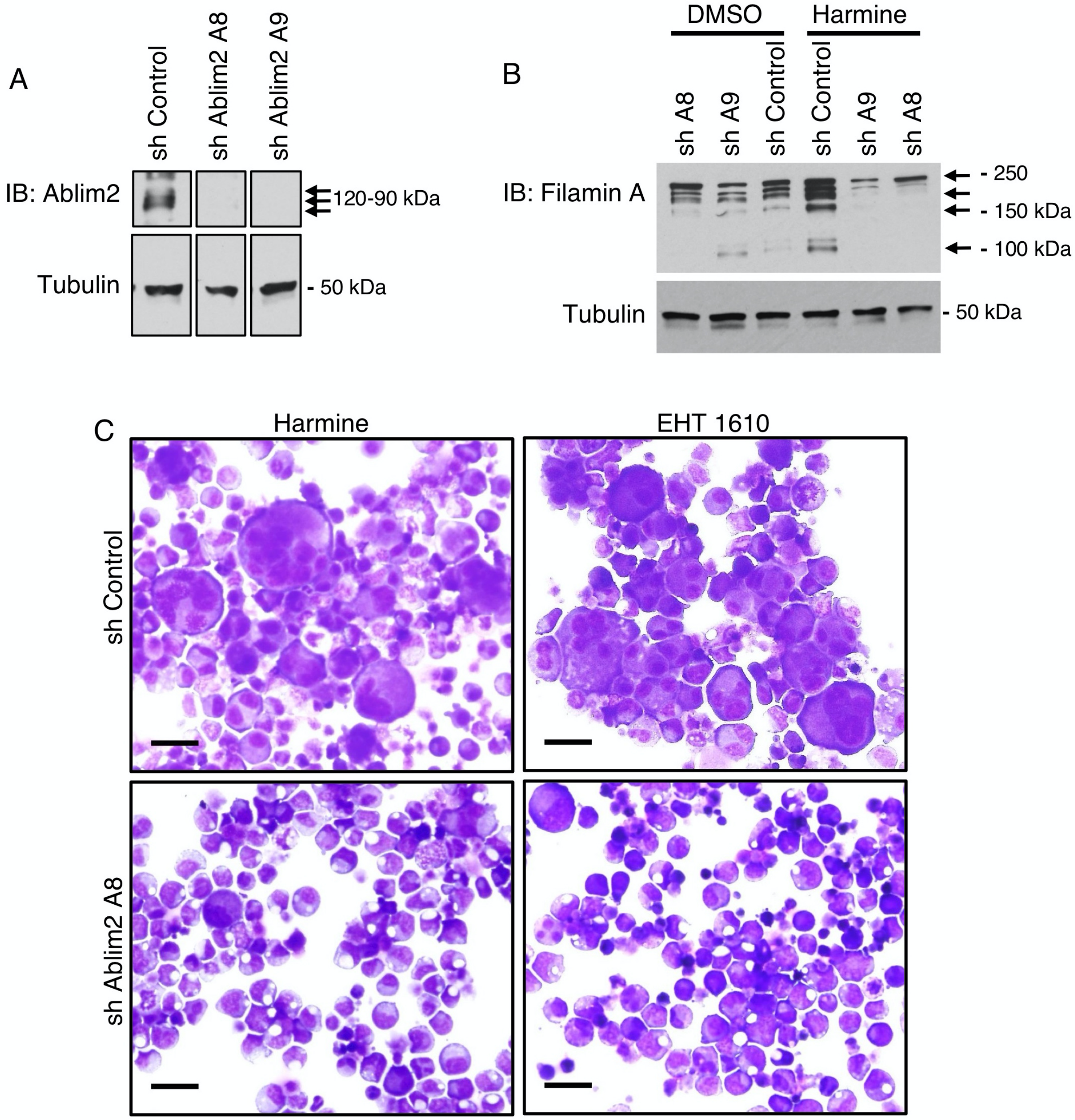

Supplemental Figure 10. Ablim2 is a critical upstream element in megakaryocyte (Mk) morphogenesis signaling. (**A**) Extent of Ablim2 knockdown. Cord blood CD34$^+$ cells transduced with control or *ABLIM2* targeting lentiviral shRNA constructs underwent IB of whole cell lysates. Results shown are representative of 2 independent experiments. Arrows: Ablim2 isoforms. Note that the three lanes are non-contiguous but all derive from the same gel-exposure (full unedited gels provided separately). (**B**) Effects on the MKL1 target Filamin A. Cells transduced as in **A** underwent 5 days Mk culture ± 5 μM harmine, followed by IB of whole cell lysates. Results shown are representative of 2 independent experiments. Arrows: Filamin A isoforms. (**C**) Morphology of cells transduced as in **A** and cultured 5 days in Mk medium ± 5 μM inhibitors. Light microscopy of Wright-stained cytospins (Olympus BX51, original magnification x 200, scale bar 20 μm). Results shown are representative of 2 independent experiments.

Supplemental Table 1. Genes from Fig. 5C regulated by stage, Dyrk, and MKL1 (shared in pink)

Up Adult	Up Dyrk Inhs	Down Adult	Down Dyrk Inhs	MKL1 Target	Up Dyrk Inhs
KIFC3 540079	GNB2L1	GPAA1	KIFC3 541240	CNN2	MYL9
HNRNPA3	HNRNPA3	KIFC3 541240	GPAA1	CYR61	CYR61
SF3B2	GTF3A	BCS1L	SCRN1	FLNA	ILK
MLEC	CENPT	PKM	PTP4A3	ILK	CNN2
CNN2	CNN2	SERPINB6	BCS1L	INPP5A	MYO1E
DNAJB6	DHX33	PTP4A3	AIF1	LIMS1	PDCD6IP
RPS3	RPS3	BID	BID	MYADM	LIMS1
MUM1	MLEC	OSBP2	BASP1	MYL9	TPM2
BDNF	KIFC3 540079	DDX46	TUBB	MYO1E	TPM1
SAMD4B	GATA5	ZNF551	PIK3CG	PDCD6IP	TPM4
GTF3A	TPM4	STAP1	SERPING1	PTGER4	TIMP3
PITPNC1	MGLL	TUBB	MAP1A	SAMD4B	SAMD4B
MYOM1	SAMD4B	KEL	TMEM173	TIMP3	INPP5A
ZFAND2B	SF3B2	BASP1	GSTM4	TPM1	MYADM
TPM4	SNORA4	DOCK2	KEL	TPM2	VASP
RBM4	PITPNC1	UBLCP1	NPRL2	TPM4	PTGER4
COMTD1	MYOM1	TRMT6	ZNF551	VASP	FLNA
GATA5	BDNF	PIK3CG	RFNG	ZEB2	ZEB2
TMBIM1	DNAJB6	ARHGEF6	DDX46	AACS	VEGFA
SNORA4	COMTD1	PDE6G	TRMT6	ABCE1	FAM98A
CENPT	GAPDH	CDCA3	UBLCP1	ACSL4	IGFBP5
DHX33	ZFAND2B	TPM3	ASCC2	ACTA2	LOXL3
GNB2L1	MUM1	RFNG	SERPINB6	ACTB	GNB2L1
UBXN1	UBXN1	PTPN22	TPM3	ACTG1	FAM43A
MGLL	TMBIM1	ASCC2	TMBIM6	ACTN1	C1orf95
GAPDH	RBM4	AP2S1	PTPN22	ACTN4	TMEM200A
MAPRE2	VEGFA	SERPING1	DOCK2	ACTR10	IGFBP2
PTGS1	FAM98A	GSTM4	DUSP6	ACTR1A	HNRNPA3
GSN	IGFBP5	PDDC1	CDCA3	ACTR3	GALNT11
LRRFIP2	LOXL3	SLC4A1	GIMAP6	ACVR1	PODXL2
DPY19L1	FAM43A	HMMR	STAP1	ADAM9	GTF3A
MYO9B	MYL9	GIMAP6	GPR35	ADORA2B	TXNDC11
PSMC4	C1orf95	ARL6IP4	PDE6G	AFF1	APLP1
FYB	TMEM200A	EGFL7	PDDC1	AFTPH	CENPT
RTN1	IGFBP2	SCRN1	OSBP2	AGPAT4	PSAT1
SIN3A	GALNT11	SDHC	AP2S1	AHCYL1	P3H3
NDRG3	CYR61	NPRL2	SLC4A1	AI846148	USP8
DBNL	PODXL2	ASH2L	DSCC1	AIG1	RASGRP1
MKRN1	TXNDC11	DSCC1	ARHGEF6	AKAP1	MAPKAP1

GPAA1	APLP1	RP11-56F10.3	SLC40A1	AKR1E1	PPP1R14A
ZDHHC7	PSAT1	XRCC3	SDHC	ALPK1	CSNK1E
TJP2	P3H3	SLC40A1	HMMR	ANGEL2	IMP4
TBC1D14	ILK	DUSP6	RP11-56F10.3	ANKRD1	NT5C3A
PRR34-AS1	USP8	MAP1A	ASH2L	ANO6	SNHG7
LGMN	RASGRP1	TMEM173	EGFL7	ANXA1	ID3
NACA	MAPKAP1	TMBIM6	XRCC3	ANXA5	ANKHD1
ACTR10	PPP1R14A	AIF1	PKM	ANXA7	DHX33
DNAJB2	CSNK1E	GPR35	ARL6IP4	ARC	FTH1
TPGS2	IMP4	HBG2	YIF1B	ARF2	NAA20
ARHGAP21	NT5C3A	MAPRE2	ASAH1	ARHGAP23	GCH1
TSNAX	SNHG7	DNMT3B	RPL36	ARHGAP29	HNRNPA1
YY1AP1	ID3	FCER1A	PBX1	ARID5B	ZPR1
HNRNPM	ANKHD1	TRAP1	STK3	ARIH1	PAPOLA
LTBR	MYO1E	TPST2	PDCD6IP	ARL10	SETD7
ADCY3	FTH1	CTC-575D19.1	CXCR2P1	ARL4D	MIPEP
FAM96B	PDCD6IP	PPP1R18	ARHGAP9	ARL6IP5	GALNT14
PDF	NAA20	DDX39B	SUCNR1	ARPC2	ZSCAN32
TNRC18	GCH1	SKIV2L2	LGALS12	ARPC4	SNX30
NLRC5	HNRNPA1	MCU	CCR4	ARPC5	GNL3
RDX	ZPR1	CPA3	CD4	ATF3	PES1
TMSB4Y	PAPOLA	GSTM1	SHROOM4	ATF7IP	SLC39A10
RPS6KB1	SETD7	DHX16	CLEC2B	ATP10A	RPS3
LIMA1	MIPEP	TPGS2	SLFN14	ATP6V0A1	snoZ196
DENND4A	GALNT14	MCM4	DOK2	ATP8B4	CXCR4
CETP	ZSCAN32	TECR	FXYD5	ATXN1	PMM2
GAS2L1	SNX30	PPP1CA	RAB38	AURKA	MLEC
VWF	GNL3	RP13-1032I1.10	SCML2	AW549877	SPNS2
CCNB1IP1	LIMS1	IGF2BP3	VNN1	B230312A22RIK	NRSN2-AS1
C11orf57	PES1	MICU2	FAM46C	B4GALT1	NT5DC2
ASCC1	SLC39A10	SPG20	OCIAD2	BACH1	CNN1
ALOX12	snoZ196	EEF1B2	ICAM2	BACH2	SLC30A5
HMGN1	CXCR4	COLGALT1	MAPKAP1	BAG2	KIFC3
VPS39	PMM2	PDCD4	SIRPA	BC031353	GATA5
PRIM1	SPNS2	NARS	YPEL3	BCAR1	MARS
KIAA1468	NRSN2-AS1	KMT5A	APOBR	BCL10	SPSB4
CD74	NT5DC2	DLK1	CX3CR1	BCL2	FAM19A5
VEGFC	CNN1	RBM34	TYROBP	BIRC6	AKAP12
C11orf71	SLC30A5	TSNAX	RP11-284F21.10	BOK	CBSL
RAMP1	MARS	NCAPD3	SPARC	BRE	EGFL6

C7orf49	SPSB4	SELO	SNRNP200	BRI3BP	SOS1
PLEK	TPM2	RPS6KB1	PGM1	BRWD1	LRRC75A-AS1
GDI1	FAM19A5	GYPE	NCF1	BTG2	CD320
SLC43A3	AKAP12	MED28	CD40LG	C130039O16RIK	GATM
USP33	TPM1	CDK1	NCKAP1L	CALU	C5AR1
ATE1	CBSL	RFC4	NCF4	CAMK2G	TMEM33
AKAP8	EGFL6	SERF2	TWSG1	CAMKK2	KCNMB1
MT-TL2	SOS1	PSME1	USP20	CAPN2	ACTG2
PTPRJ	LRRC75A-AS1	MRPL42	HCST	CAPZB	C11orf96
F13A1	CD320	ELOVL5	NCF1C	CAR9	TSPAN2
RPL35A	GATM	NOTCH2NL	GLRX	CAV1	MGLL
NONO	C5AR1	TPSAB1	PLIN2	CBL	COTL1
RPP30	TMEM33	MKRN1	MVP	CBX4	NRARP
FXYD5	KCNMB1	HNRNPK	BNIP3L	CBX6-NPTXR	CTR9
DNMT1	ACTG2	SNX5	PTGIR	CCDC45	PSMC5
SUPV3L1	C11orf96	LAMTOR5	HPRT1	CCDC85B	GRHPR
CTTN	TSPAN2	RPLP0	CCRL2	CCNL2	SLC27A4
EGLN2	COTL1	LMNA	KCNN4	CCRN4L	GATA2
GUCY1A3	TIMP3	TSEN15	GDI2	CDC26	PLPP3
NANS	NRARP	RUVBL2	PSAP	CDC42BPA	CPNE1
CAST	CTR9	CFAP97	FNBP1L	CDC42EP3	SF3B2
NPDC1	PSMC5	MATK	ZNF185	CDC42SE2	WNT5A
SUMO3	GRHPR	LCP1	SLAMF6	CDC7	DAP3
CIPC	SLC27A4	FAM171A1	MEIS3P1	CDK17	STAT5A
FMNL3	GATA2	IGF2BP1	FN3K	CDKN2C	LRRC75A
SSBP2	PLPP3	SLFN13	MYO1F	CFL1	SH3PXD2B
NAA38	INPP5A	RPS11	N4BP2	CFL2	SNORA4
SELO	CPNE1	KCNH2	CPXM1	CHCHD2	DDX50
MTMR10	WNT5A	DAP3	ELMO1	CHEK2	CTXN1
NBPF14	DAP3	SEPT7	PTPRE	CHST11	BSPRY
SPARC	STAT5A	ATP5J	ITM2A	CIRBP	METRN
NOP9	LRRC75A	SUOX	GRHPR	CLDN12	PITPNC1
MAN2C1	SH3PXD2B	KMT5B	MFSD1	CLTB	TTC3
HLA-A	DDX50	SAMSN1	CLCN3	CLTC	AOAH
CDK14	CTXN1	BORA	MLH1	CNN3	ROBO3
GK	BSPRY	RNASET2	MEI1	CNOT3	HBD
MIR4701	METRN	SLC39A7	KCNQ4	CNST	MYOM1
MAOB	MYADM	BCAS2	DPYD	COBLL1	KALRN
PSMB9	TTC3	NUDT4	RPS6KA1	COL12A1	PCSK1N
MT-TS2	AOAH	POLR1D	TMUB1	COL5A1	IFRD1

KRI1	ROBO3	BIVM-ERCC5	ZNF410	COL5A2	MRVI1
PPBP	HBD	RPSA	AP001189.4	COL6A2	GNAS
EML4	VASP	ITGA4	RHD	COL8A1	C14orf159
BANP	KALRN	ARRB2	CUX1	CORO1C	ZNF692
RBM22	PCSK1N	UCKL1	PLAG1	CPNE2	SHMT2
TBRG1	IFRD1	BRD8	MGST2	CPT2	RPLP2
POLR2J3	MRVI1	ATF7IP	NYNRIN	CREB1	RNASEH2B
AKT1	GNAS	TRADD	NARFL	CREBBP	POM121C
ACTN1	C14orf159	XIST	PSRC1	CRIM1	TNFAIP3
IFI44	ZNF692	FLII	TSPAN33	CRK	BDNF
PPFIA1	SHMT2	NEMF	WBP2	CSNK1G2	PPRC1
CLEC1B	RPLP2	CDC25C	NAGK	CSRP1	TFAM
SCARF1	RNASEH2B	RPL10	ABCD3	CSTF3	ECE2
CCND3	POM121C	CDK5RAP1	LYZ	CTDP1	DNAJB6
SNW1	TNFAIP3	HSPA8	C3orf38	CTGF	POLR2G
ACSL5	PPRC1	CLN3	GRAP	CTNND1	REC8
SLC44A1	TFAM	OXA1L	MAP3K8	CTPS	EPHB4
GNAZ	ECE2	SLC43A3	MAP4K1	CTPS2	TIMM8A
CCDC71L	POLR2G	ITGB2	C15orf57	CTTNBP2NL	RPLP0
CNPPD1	REC8	SV2A	PTPN6	CUX1	ULK3
SMARCB1	EPHB4	REPS1	RP11-879F14.3	CYFIP1	NUDT11
PVRL1	TIMM8A	NBPF14	NDFIP1	CYTSB	MTFP1
NXF1	RPLP0	RPL34	ATAD2B	D18ERTD653E	ZC3H8
SCN1B	PTGER4	NLRC5	RECQL	D4BWG0951E	RPL19
DNAJC16	ULK3	GYPB	MLLT11	D5ERTD579E	AUP1
MEN1	NUDT11	TMEM14B	UPK1A-AS1	DAPK3	VIM-AS1
MAT2B	FLNA	ZDHHC4	TMEM14A	DARS	CENPV
TIMM44	MTFP1	RSU1	TPM4	DCUN1D3	MTHFD2
TP53INP2	ZC3H8	AHSP	DUSP14	DDAH2	PITPNM1
DIAPH1	RPL19	BCAT1	UBE2E3	DDHD1	PIGT
TRBV7-5	AUP1	TYW3	ZNF112	DENND4B	AC008079.10
NARF	VIM-AS1	GAPT	VTA1	DMWD	COMTD1
DENND2D	CENPV	TBRG1	CD300LF	DMXL1	GAPDH
RPL36A	MTHFD2	ZFX	CYBA	DNAJB4	PFAS
KCNA3	PITPNM1	SNRPB	HMGB1	DNM3	RPL17
FHOD1	PIGT	MRPL43	PPIL3	DOT1L	TYSND1
HYPK	AC008079.10	TMEM261	KANSL3	DSCAM	HSPA8
EXOC7	PFAS	DHX35	NUCB2	DSTN	PRR22
NCAPD3	RPL17	PSAT1	DEF6	DTNA	FRZB
SLC50A1	TYSND1	COL4A5	DERL1	DTX1	SLCO4A1

MKRN2	HSPA8	CKB	SNX20	DUSP1	PSMD4
ARHGEF10	PRR22	MEN1	TXNIP	DUSP5	CNBP
CSNK2B	FRZB	NLRP2	CAPN2	DUSP6	TNF
RP11-588K22.2	SLCO4A1	HAUS5	KIAA0930	DYRK1A	GADD45A
MYL12B	PSMD4	TARS	LDHD	E130311K13RIK	USP36
CD276	CNBP	NME7	FBXW4	ECM1	RPS18
INTS8	TNF	TNRC18	SLC9A3R1	EEF1B2	XXYLT1-AS2
INF2	GADD45A	INPP5F	ARRDC3	EFEMP2	RAC3
NCK1	USP36	IDH3B	NUP85	EGFR	PFKFB2
CCHCR1	RPS18	COX6C	SH3BGRL	EGR3	ZFAND2B
MFSD2B	XXYLT1-AS2	RP11-673E1.3	LPAR5	EHBP1L1	MUM1
MT-TG	RAC3	LIN28B	RSAD2	EHD1	MRPL2
ATP5C1	PFKFB2	RBCK1	CD36	EHMT2	BTN2A1
ALDOA	MRPL2	CCBL2	MRPL51	EIF2S2	UBXN1
C11orf24	BTN2A1	RP11-651P23.4	ACP1	EIF4A3	HACD1
44809	HACD1	ARAP1	SDCBP	EIF4H	BCS1L
SEC13	BCS1L	CFH	IFIT3	ELK4	NAF1
NFE2	NAF1	ZSCAN29	DHX8	ELL	IFRD2
NBR1	IFRD2	THOP1	SAV1	ELL2	HMG20B
MANBAL	HMG20B	GRAMD1A	SAMD9	ELOVL5	RAN
SYNM	RAN	CENPM	PDGFC	EML4	RPL15
Y_RNA	RPL15	PPP1R7	MSANTD3-TMEFF1	ENC1	B4GALT3
ABR	B4GALT3	NADK2	SLA2	EP400	HNRNPH1
ARL13B	HNRNPH1	CHTF18	PRMT2	EPB4.1L3	RPL27
PRKG1	RPL27	MIS12	ZNF81	EPHA1	RHBDD3
DGKZ	RHBDD3	C1orf186		ERC1	ITGA6
ABCA7	ZEB2	CNRIP1		ERC2	CPSF3L
PSRC1	ITGA6	KLF1		EREG	TMBIM1
PTGIR	CPSF3L	GRAP2		ERI2	RBM4
R3HDM4	RCN2	HGS		ERRFI1	RCN2
PYCRL	CCDC107	ZNF277		ESYT2	CCDC107
TMSB15A	RPUSD1	ZNF687		ETF1	RPUSD1
BOD1L1	HSF1	NCAPG2		ETS2	HSF1
SARM1		GSTM2		EXD2	
CXCL3		SEPT9		EXOC4	
ATP9B		ACOT8		EXOC5	
ATP2A3		UHRF1		F2R	
CD9		MTA1		F3	
SOD2		RPS2P5		FAF1	
ACTN4		NUBP2		FAM100A	

GSAP	EIF3G	FAM113B
SUMF2	AKAP2	FAM118A
ESAM	WDR1	FAM126B
ASAP2	FBLN2	FAM134C
SPSB1	CASS4	FAM179B
CMTM5	DDX56	FAM188A
PRICKLE2	SERPINH1	FAM198B
PIK3C3	ZBTB14	FAM3C
PFN1	CRYGD	FAM53A
LTBP1	ALDOA	FAM60A
LONP2	ATG4C	FARP2
THBS1	RAC3	FAS
TCF3	EML3	FAT1
ITGA2B	CUTA	FAT4
TRBV7-4	DDIAS	FCHO2
UBA7	B3GAT3	FERMT2
ZNF467	FPGS	FERT2
STRN4	CCNH	FEZ1
CXCL1	PTK2	FGD3
IL6R	FAM173B	FHL2
ADD3	C14orf2	FHL3
TCTN3	NCEH1	FKBP10
LTC4S	EZR	FLNB
LST1	ALDH16A1	FLRT2
LPIN2	CAPG	FOSB
MCCC1	HK1	FOSL1
EBAG9	DNAJC17	FOSL2
NMD3	FSCN1	FOXF2
RGS3	PYROXD1	FOXP1
ACACA	HNRNPM	FRMD4A
ANO10	DDX11	FRMD6
TMEM259	PABPC1	FSCN1
ATG4A	PYCRL	FYN
MT-TH	ZMYND11	GAB2
OSBPL9	CNN3	GADD45G
SNX13	BACE2	GALK1
PIGS	MYH10	GATAD2A
CCNC	SLC25A22	GBF1
NCK2	PUS3	GCNT2
PSMG2	UBE3A	GDAP2

TBXAS1
DDO
HDAC1
SNRPA1
LAMP5
RMND5A
MAX
FAM89B
ADCY6
DPP4
MPP1
ANKRD12
PSTPIP2
LAT
ANAPC7
CTD-2260A17.2
GOLGB1
MMP17
RABEP2
PDLIM5
STX16
YIF1B
EGLN3
TNS1
MCMBP
SUPT5H
RAD51AP1
GTPBP2
HOOK3
PDE3A
PRR7
UBFD1
ANKRD10
NR3C1
TM6SF1
ATP5I
SCRN3
SDHA
AIG1
TAF4
CTD-2192J16.15
GYPC
ABCF3
MCM10
EML4
POLA2
TBC1D23
EIF5A
YY1AP1
WIPI2
LRSAM1
NLE1
STRADA
C11orf49
PIR
SSR2
FZD6
PDP1
USP39
HSD17B11
NSUN4
SYNGR3
ARID3A
TJP2
PSMD1
RNF144A
CERKL
RPS17
RPS13
TMIGD2
RDX
USP11
RHAG
STAG1
PKLR
CDKN3
FAM178B
PCNT
SASH3
GEMIN2
GFOD1
GGA3
GHRL
GLIPR1
GLIPR2
GLRX
GM10094
GMDS
GNPDA1
GPAM
GPR149
GPR153
GPR176
GPSM2
GRB14
GRB2
GRK5
GRK6
GRN
GSPT1
GTPBP1
GTPBP3
GUSB
H3F3B
HDAC4
HES7
HIF1A
HIVEP3
HPS4
HSD17B12
HSD17B7
HTRA1
IER5
IGF1R
IGFBP7
IPO7
IRF2BP2
ITGA1
ITGA5
ITGAV

RP11-81H14.2	METAP1	ITGB1
SALL2	SAAL1	ITGB3
MDM1	CDC123	ITPR1
CD68	RPL32	ITPRIP
AC004069.2	LTB	JRK
CTSA	MAN2C1	JUN
STRIP2	AMN1	JUNB
OLFML2A	ABR	KANK1
TBC1D31	CORO1C	KCNK7
ELMSAN1	METTL10	KCTD10
H3F3B	H3F3B	KCTD5
AC010084.1	CCNB1	KDELC2
NES	DDB2	KHDRBS1
SMG1P1	TRIP6	KIF5B
NADSYN1	RABEP2	KLF13
CEP44	SCARB1	KLF16
BTK	ADGRA3	KLF6
NOL11	NXT2	KLF7
AC022182.3	GORASP1	KLF9
COL24A1	ARID3B	KLHDC8A
PDGFB	SUMF2	KLHL28
FYN	ANAPC7	KRR1
MDK	HMGA1	LASP1
SLC35D3	PTK7	LASS5
PKIG	DDT	LASS6
DEDD2	NMNAT3	LATS2
WHAMMP3	USPL1	LBH
STXBP2	GAR1	LEMD3
RP13-890H12.2	SNHG5	LEPROTL1
FNIP1	PPP4C	LIMA1
ACTR1B	SPRTN	LIMK1
HOMER2	ECI1	LMNA
TCEA1P2	MARCKSL1	LOX
ARRDC4	EIF2B4	LPAR4
ALDH16A1	C12orf57	LPHN3
PGM1	PABPC4	LPP
SIMC1	EDRF1	LSM11
NEXN	TRIM8	LY75
XX-FW83128A1.2	TCEB3	LYRM1
GSTO1	PTER	M6PR

NOG	ALG3	MAFK
ZNF213-AS1	PRDX2	MAML2
TMEM214	ACADM	MAN2A2
SKIV2L2	MRPL30	MAP2K3
TCF12	ASNA1	MAP2K4
SACM1L	THOC3	MAP3K11
CCT6P1	IFT27	MAP3K15
ZNF185	GINS4	MAP3K8
NAT1	DIS3L	MAP4K3
SLC22A17	CCDC3	MAPK1
EHMT2	PHGDH	MAPK14
CXCL2	RP11-706O15.5	MAPKBP1
MECR	SKIL	MAPRE1
HERC2P2	HEMK1	Mar-09
C2orf88	FAM213A	MAST4
PTPN6	CPSF3L	MAT2A
DGCR14	FBXO30	MBNL1
LUC7L3	CD48	MEAF6
PRKAA1	BAX	MED15
S100A10	APOE	MED21
VPS13D	CUL7	MESDC1
RP3-370M22.8	PLTP	MFSD3
KDM5D	ADA	MFSD7B
JMJD6	MYB	MGA
TRIM7	MTFR2	MICAL2
MON1B	DUS2	MID2
ATP8A1	WDR36	MLL3
KAT2A	KCNQ5	MMP9
RTP4	INTS8	MOBKL2A
ZCCHC11	RNMT	MORC4
CDAN1	MAP2K4	MPDU1
EML2	RPS3A	MRGPRF
NPIPA1	RP11-620J15.3	MRPL51
RAP1B	PIM2	MRPS17
PLCB2	ST7	MRPS7
SNX21	ASNSD1	MSN
PYGL	H2AFY2	MSRB3
ATP6V0A1	MYO1G	MTAP1B
GTF3C5	RPL13A	MTHFD1
FRS2	ROGDI	MTMR14

C4orf36	NDUFC1	MXRA8
ABCC3	AURKAIP1	MYBL1
HNRNPUL1	TNFSF9	MYBL2
RAB34	BLVRB	MYH9
AKT1S1	ICAM4	MYL12B
PRKY	GNPDA1	MYO1C
NIF3L1	ZBTB44	NBN
CDC42BPB	FGFR1OP	NDUFS1
GCC2	AIG1	NEDD4L
PKD1P6	MECOM	NEDD9
RNF8	FADS2	NFIL3
ALOX12-AS1	C11orf73	NLGN2
SMAD1	PAFAH1B3	NPAS3
ISCA1	PLXNC1	NPAS4
GABBR1	FAM89A	NPPB
MAML1	TIMP3	NPSR1
BACE2	TMEM199	NR4A1
FYCO1	PUM3	NRBP1
CAMTA1	PIM1	NUP155
SLC2A3	DTYMK	OAT
ZNF385D	CCT6P1	OGDH
TBC1D3L	ZNF138	OGT
PDGFD	GOLM1	OPN1SW
CTNNB1	RNH1	OTUD4
TTYH3	NRF1	OXCT1
C6orf25	RBM42	OXSR1
ALPK1	TSPAN13	PALLD
SLX1A-SULT1A3	RPS4X	PALM2
TSPAN32	AC002454.1	PARP8
HTR2A	SAT2	PAWR
ACTG1	HOXB2	PCBP2
ADRBK1	CFL1	PCF11
PXDC1	OMA1	PCGF5
ANKZF1	PRSS57	PCNP
FAM150B	DCTD	PDE4DIP
LHFP	MRPS14	PDHA1
ZNF584	CXXC5	PDIA6
ARHGAP10	IMMP1L	PDLIM5
CEP290	IQGAP1	PDLIM7
CD99	IGF2R	PDPK1

RP11-676M6.1	RASGRP4	PELI1
IRS2	BIRC6	PER1
LMAN2L	OSBPL6	PER2
MTFR2	UBE2G1	PFKFB3
STX11	GEN1	PHLDA1
KMT5C	PTPN13	PICALM
MSN	NOL4L	PIK3CB
ZNF619	NPL	PIP5K1A
CBFA2T2	SNX17	PIP5K1C
PSMG4	RNF144A-AS1	PITPNA
PFKM	NADSYN1	PJA2
MSH5	IFRD2	PKD1
HERC2P3	MEG3	PKP2
RBM39	AP1B1	PLA2G4A
RP11-159G9.5	GALK2	PLA2R1
GPX1	USP47	PLAU
CUL4A	TFRC	PLEKHB2
NBPF11	RAD51AP1	PLEKHG2
TUBA1C	LAMC1	PLEKHO2
SSH3	TNRC6C-AS1	PLK3
PHF21A	SPIN1	PLRG1
TMEM222	SLC25A6	PLS3
EME2	IGFBP4	PNMA1
THOC3	ISYNA1	PPAP2A
TNFAIP2	ASB9	PPARD
CDCA3	GALM	PPIB
ITGB3BP	KCNK17	PPM1H
HERC2P9	SLC16A10	PPP1R12A
LINC00853	FASTK	PPP1R12C
LINC00211	CTNND1	PPP2R3A
PPFIA3	TMEM216	PPP3CB
NDUFS5	INO80E	PPPDE2
MAP3K10	EIF3E	PRC1
KRT8	IP6K1	PREPL
SEC61B	HDHD2	PRG4
STAU2	SPG21	PRIM2
MBIP	MKL2	PRKAA1
MYO15B	SCNM1	PRKAB2
NAT14	VIMP	PRKAG2
COX5B	SLC4A2	PRKCC

SBDSP1	GMPPA	PRKD1
TBC1D23	HMGCS1	PRRX1
THBS3	VSIG10L	PRRX2
VPS28	TUBB2A	PTGS2
COMMD6	TMEM161A	PTPLA
WHSC1L1	NCAPH2	PTPN14
SAV1	ID1	PTRF
DHX35	KIFC1	QPCTL
C17orf62	ANAPC5	QSOX1
CDC25C	ITGB3BP	RAB11FIP2
CMPK1	RHOC	RAB11FIP3
ZSCAN26	RP11-111M22.3	RAB21
ATG13	ZFAND1	RAB23
DNM1L	SON	RAI14
SHC1	DHTKD1	RALY
NR2C2	LINC01218	RANBP2
SET	TUBGCP2	RAP1B
TEP1	SLC25A26	RASA4
ABO	PPA1	RASSF2
GBA2	IST1	RBBP4
KLHL36	HMGB3	RBBP7
QPCT	CCT8	RBMS3
MMP24-AS1	SAE1	RDM1
TMEM147	CNST	RELB
COG1	KBTBD8	REV3L
GOLGA8R	MRPL49	RFWD2
AC007383.3	ADSL	RFX7
AKIP1	DDAH1	RGL1
FAM127C	MEF2D	RHEB
STK16	STMN1	RHOB
MGAT4B	EMP3	RHOBTB3
ZFPM1	HMBS	RLF
PPP1R15A	BIN1	RLIM
TMEM219	EFNA4	RND3
RERE	CEP57L1	RNF11
RPL27A	TBC1D8	RNF19A
VOPP1	FAHD2B	RNF216
GUCY1B3	TIMM9	RNF34
RCAN1	NCK1	RNFT1
CYLD	DENND5A	ROCK2

CNST	FANCI	ROR1
NAP1L4	RPL18	ROR2
AP000476.1	COPS3	RPA1
APEX1	SEC24C	RRBP1
TM7SF2	ZNF227	RSU1
RABGAP1L	TLE1	RTN4
NIPAL3	SMIM13	RUNX2
LSP1P2	SPI1	RUSC2
ARHGAP6	NQO1	S1PR3
MAPK3	C17orf100	SAMD4
ICAM2	FTH1	SAP18
TGFB1I1	POLR3E	SCYL1
MLLT6	RPL8	SCYL2
RUFY1	PYCR1	SDC2
PSMD6	NMRAL1	SDC4
PIP4K2A	DCP1B	SDPR
CASP3	B9D1	SEC24D
PLPP5	RPARP-AS1	SEMA3A
RBPMS2	RPUSD3	SENP3
CLEC12A	SYNGR2	SEPHS1
PIDD1	DBF4	SERP1
MPP5	RP11-47A8.5	SERPINE1
AMH	EPHB4	SERTAD1
FAM8A1	LMBR1L	SERTAD2
SAT1	SNAPIN	SETBP1
DPPA4	SMYD5	SFXN3
ULK4	FBL	SGCD
NAGPA	TEX261	SGK1
WASH7P	ECHDC1	SGMS1
DDX11-AS1	C16orf59	SGTB
FAXDC2	TMEM256-PLSCR3	SH3BGRL
SF1	NFX1	SH3GL1
NBR2	FAM192A	SH3GL3
BANK1	MEAF6	SHB
SNRNP70	IPO11	SIDT2
ZNF511	ILF2	SLBP
TRMT1	SPAG7	SLC10A6
DNTTIP2	ALDH1A1	SLC10A7
MAST4	BTF3L4	SLC25A19
NUBP2	NAPA	SLC2A1

NPIPB3
AL022344.7
SQRDL
AC005083.1
INPPL1
TRHDE
SLMAP
DUX4L50
RAB11FIP3
SELP
SSR1
MMP14
RHOQ
RP4-816N1.6
NUCB2
EIF4G3
RAP1GAP2
TNFRSF14
FAM109B
SPTB
HERPUD1
H1F0
CUEDC2
ATG4C
CFL1
ZNF837
RGS14
DAPK3
UNC119
CGGBP1
UTP18
MIR6753
PPIA
NME4
PLCXD1
DNM3
XRCC3
LSM5
CLIC2
RP11-713M15.2

NAP1L4
UBE2E2
NIPSNAP1
NAE1
RPS2
CA5BP1
RPL37
EMC1
RPL7
POMT2
ZWILCH
CBWD1
PMM1
CTD-2116N17.1
SLC50A1
RPLP2
APEX1
GATB
CENPH
POC1A
SET
ZG16B
LINC00665
TBC1D31
RP11-301O19.1
ATXN7L3
AMMECR1
PUDP
DUSP2
MCRS1
MUM1
CTH
EIF3K
RSBN1
SLAIN1
PARVG
SLC25A3
CDK6
MICALL2
PRDX1

SLC3A1
SLC6A9
SLC7A1
SLCO3A1
SLMAP
SMAP2
SMG1
SMNDC1
SMURF2
SNAI2
SNX18
SNX33
SNX6
SORBS1
SOX6
SRC
SRF
SRFBP1
SRGAP2
SSFA2
ST5
ST7
STAMBPL1
STARD13
STAT3
STK38L
STK40
STX11
STXBP1
SUFU
SYDE2
SYN3
SYNGR2
SYNPO2
SYS1
TACC2
TAGLN
TATDN2
TCEB1
TCF20

ZC3H12A	VIM	TCTEX1D2
ALS2CL	RPS14	TEAD1
PCDHGB5	EMC7	TEC
AAED1	BTRC	TES
SLC25A15	C19orf25	TFB2M
VSIG10L	AZI2	TGFB1I1
KMT5B	GSTZ1	TGFBR3
FAM162A	ZNF562	TGOLN1
PARP15	TPCN1	TGOLN2
CIRBP	CMC4	THBS1
ICAM5	DGAT1	TIFA
MYH9	SLC25A17	TJP2
ASPSCR1	RTTN	TLE4
ZNF800	CCDC50	TLN1
CCDC66	TAF15	TM2D2
HPCAL1	RPP30	TM4SF1
BIVM	RWDD1	TMED9
SGF29	NPC2	TMEM107
RP11-930O11.1	ASB8	TMEM123
C19orf43	NDRG1	TMEM183A
ATG14	NUDT5	TMEM47
GATAD2A	HSP90AA1	TMPO
CACNB4	MCCC2	TMSB4X
C11orf58	UAP1L1	TMTC3
EAF2	PDCD5	TNC
ATN1	PRG2	TNK1
PIGQ	SLC25A4	TNK2
SNX19	RBFOX2	TNRC18
TMEM40	ZBTB10	TNS3
TMEM63A	PTGDR2	TPCN2
IPO4	GLUL	TPST2
PPP4C	ZNRD1	TRAF3IP2
OAZ1	IDH3A	TRAF5
CD37	TPRKB	TRAP1
ZSCAN2	MAP4K2	TRERF1
RP11-282O18.3	C1orf109	TRIB1
ATP5B	BCL7A	TRIM16
SPPL2B	SEH1L	TRIO
PSMC3	FKBP3	TRP53BP2
ZBTB4	UQCRFS1	TSC22D2

BAP1	TTF2	TSGA14
TRMU	TMEM117	TTC28
AIP	TMEM251	TTLL11
WDR62	MECR	TTLL3
CHSY1	IARS	TUBA1C
HLA-B	TELO2	TUBB5
RNU4-52P	PDCD2L	TXNRD1
ACOT8	TMEM184B	UBC
EIF4G2	ETV4	UBE2Q2
MAP3K5	SLC19A2	UBE2W
RAC1P2	TANGO6	UBE4A
IRAK1	JAZF1	UBFD1
TCEANC	S100A11	UBR4
PON2	FUOM	UGP2
BPTF	CYYR1	UHRF1BP1L
RPL13AP25	CDKN2C	UNC45A
RP11-154D6.1	NUP98	USE1
TLK1	SH2D2A	USP2
LAMTOR2	CERS4	USP24
INTS9	CYB5A	USP32
FAM160A2	C3orf33	USP37
UACA	PIGO	USP4
FKBP15	SNHG1	UTRN
TPD52	RPL10A	VASH2
SNHG11	ZPR1	VAV3
ADCY9	PSMB2	VCL
NCOR1	HLA-DMA	VGLL3
C5orf56	LDHA	VIM
BTBD11	SLC25A5	VPS33B
ODF2	GPR85	WDFY1
SLX1B-SULT1A4	RP5-1198O20.4	WDR1
HIVEP2	RAPGEF1	WDYHV1
MAPKAPK3	LST1	WEE1
DKK1	ZNF74	WIPF1
CPSF1	LSM6	WRB
AC073254.1	RAP2A	WWTR1
PPM1L	HNRNPF	XRCC6BP1
LYL1	CH507-254M2.1	YAF2
SRRM2	MUC1	YIPF1
SMIM6	CHCHD4	YKT6

NFAT5
TMEM246
FCHSD1
AGAP3
RPS25
MAP3K7
GTF2H1
DCAF5
AP1G2
SEMA4D
SLC25A39
PKD1
TP53I11
WBP2
CEP131
SPAG7
FAM193B
NEURL1B
WDTC1
NCKIPSD
PAQR3
FGFRL1
ENY2
LSM7
MRPS7
SNORD58A
RPS20
MICALL2
ITGA9-AS1
CEP85
SBDS
AC002310.14
RP11-867G23.4
ADGRG1
EOGT
DGCR6
FAM98A
TMEM69
SPNS1
GNL2
RGS14
LRP10
ARIH2
CCDC134
AC024592.12
DLEU2
RPL17
RTKN2
LYPLAL1
PPP1R14A
ARMC1
CCDC130
NDUFS2
HACD3
INTS4
TMEM150B
ALDH9A1
RNF130
NME1
MRPL33
CTSL
HAUS4
CEP44
CCT2
METTL1
MRPS27
TUBB6
AHSA1
EIF4A1
LRR1
EXOSC9
DUSP12
RAN
HSCB
ALG6
YWHAG
YWHAZ
ZBTB11
ZBTB20
ZC3H12A
ZC3HC1
ZDHHC14
ZFAND3
ZFAND5
ZFC3H1
ZFP143
ZFP281
ZFP326
ZFP36
ZFP521
ZFP568
ZFP644
ZFR
ZMAT1
ZSWIM4
ZSWIM6
ZSWIM7
ZYX
ZZZ3

NEK9
SH3GL3
SLC25A1
ZNF254
MRPL2
SNRNP70
TMBIM4
CCNK
TXNL1
HES6
PSMD13
CCDC138
TKT
SMURF2
NNAT
ARHGEF18
AURKB
SMIM12
NDUFA12
EEF1D
MEST
HNRNPA1
TMEM30A
EXOG
SFR1
PSMD14
HES1
PTOV1
ATF7IP2
UBE2D1
SRP14
AURKA
CIRBP
PTS
HIST1H2AC
RAD23A
CDCA7
MPP5
SYNGR1
ORC4

SNX13

CCDC167

PI4KB

DIP2A

ZNF837

THNSL1

C15orf40

PON2

NT5DC3

NBAS

ZNF2

DDX39A

FHL3

FAM19A2

TFR2

CFP

MARCH7

EXTL3

MFNG

ABHD11

NACA

STK17B

MRPL55

STMN3

SMIM19

RP11-864I4.1

ODC1

CHCHD7

MRPL3

NUP62CL

FAM86C1

WDR4

WASH6P

GALNT7

CTD-2006C1.2

VCPKMT

OGFOD1

Supplemental Table 2. Enrichment categories (Human Gene Atlas): genes downregulated both with Dyrk inhbitors and in adult versus neonatal megakaryocytes. Top 10 of 29 entries.

Index	Name	P-value	Adjusted P-value	Odds Ratio	Combined score
1	CD71+ Early Erythroid	0.000046	0.001341	7.12	71.09
2	CD56+ NK Cells	0.001985	0.02879	4.40	27.39
3	Leukemia promyelocytic-HL-60	0.1386	0.6731	6.94	13.71
4	CD14+ Monocytes	0.06406	0.6193	3.43	9.44
5	Small Intestine	0.09781	0.6731	3.94	9.15
6	Pancreas	0.2064	0.7169	4.45	7.02
7	Colorectal Adenocarcinoma	0.1552	0.6731	2.95	5.49
8	Lymphoma Burkitt’s (Daudi)	0.2848	0.7378	3.05	3.84
9	721 B Lymphoblasts	0.1625	0.6731	1.71	3.11
10	CD33+ Myeloid	0.2225	0.7169	1.90	2.86

Supplementary Methods

Mice genotyping

The genotypes of *Mkl1* knockout mice were determined by RT-PCR using primer 1 (5′ TGCTTGCATGTATGGCTGTT 3′) and primer 2 (5′ TGTTTGGTGCTCAGCAGTTC 3′) from the intronic sequences between exons 14 and 15 and primer 3 (5′ CAGAAAGCGAAGGAGCAAAG 3′) from the *neo* resistance gene; the PCR generated 340-bp wild-type and 500-bp targeted bands. The primers used to genotype for *Dyrk1afloxed* allele are forward primer 5′-ATTACCTGGAGAAGAGGGCAAG-3′ and reverse primer 5′-TTCTTATGACTGGAATCGTCCC-3′. The primers used to genotype for pF4-Cre include Cre forward primer 5’-CCCATACAGCACACCTTTTG-3’, Cre reverse primer 5’-TGCACAGTCAGCAGGTT-3’ and internal control IL-2 forward primer 5’-CTAGGCCACAGAATTGAAAGATCT-3’ and reverse primer 5’-GTAGGTGGAAATTCTAGCATCATCC-3’. PCR protocols for genotyping are described in Jackson laboratory website.

Quantitation of immunofluorescence (IF) images

IF image quantifications were performed using Fiji ImageJ version 1 (https://imagej.nih.gov/ij/). Quantification of MKL1 and Phalloidin were performed on single-plane, mid-nucleus images captured with a 63x oil immersion objective on a Zeiss LSM-700 confocal microscope. To calculate MKL1 nuclear/cytoplasmic ratios, MKL1 nuclear fluorescent intensity was measured as the mean fluorescence intensity (MFI) of Alexa-488 (MKL1) nuclear signal overlapping with DAPI signal after filling holes in DAPI signal by applying Gaussian blur (Sigma = 2); threshold was set to 86, 255 and then

converted to mask; watershed was run, and particles sized 125-infinity were analyzed; circularity was set 0.7-1.00; and all cell debris was excluded manually. The image was converted to composite, and channels were split. MKL1 cytoplasmic MFI was calculated by subtracting the mean Alexa-488 nuclear signal from the mean of Alexa-488 pan-cellular signal. The ratio was obtained by dividing the sum of the nuclear signal over the sum of the cytoplasmic signal using ≥ 50 cells per experiment. To quantitate Phalloidin (F-actin) MFI, debris was excluded manually; images were converted to composite; channels were split, and pan-cellular Alexa-594 MFI was measured for ≥ 50 cells per experiment. The sum of Alexa-594 MFI was divided by the number of cells to obtained Phalloidin MFI per cell. To calculate Ablim2 MFI, maximum intensity Z-stack projections were created and converted to composite; channels were split, and then lines were drawn around the border of GFP+ cell. Pan-cellular Alexa-594 MFI was measured inside the border. This measurement was applied for ≥ 50 GFP+ cells per transduction from 2 independent experiments.

RNA-seq for later stage neonatal megakaryocytes

Human cord blood CD34+ cells cultured for 11 days in megakaryocyte differentiation medium + 2.5 μM harmine underwent lysis, RNA extraction and sequence platform as described for the early-stage megakaryocytes (Figure 5C). RNA sequencing data was preprocessed following FastQC output to check for quality issues and compiled with MultiQC. Alignment was done using Kallisto in paired-end mode with gc-bias flag, aligning to the cDNA GRCh38 build from Ensembl [1,2]. Pseudoalignments were processed for differential analysis using tximport passing off to Deseq2 [3,4]. After

Deseq2 normalization and quantitation shrinkage was performed using approximate posterior estimation for GLM (apeglm) effect size estimation, volcano plots were generated with EnhancedVolcano [5]. Sequence files have been deposited in GEO (GSE204909).

Supplemental Table 3

List of the antibodies used for immunoblot

Antibody	Species	Company (Catalog number)	Dilution (Buffer)	Duration (temperature)
Anti-Dyrk1a	Mouse	Abnova (Clone 7D10. Cat# H00001859-M01)	1:500 (1% milk in *TBST)	Overnight (4° C)
Anti-MKL1	Rabbit	Bethyl Laboratories (A302-201A-M)	1:1000 (1% milk in TBST)	Overnight (4° C)
Anti-Filamin A	Rabbit	Bethyl Laboratories (A301-134A-M)	1:2000 (1% milk in TBST)	One hour (Room temperature)
Anti-Hic-5	Mouse	BD Transduction (611165)	1:500 (1% milk in TBST)	Overnight (4° C)
Anti-HEXIM1	Rabbit	Bethyl Laboratories (A303-113A)	1:2000 (1% milk in TBST)	One hour (Room temperature)
Anti-ACTININ1	Rabbit	Cell Signaling (3134S)	1:1000 (1% milk in TBST)	Overnight (4° C)
Anti-IGF2BP3	Rabbit	Millipore (07-104)	1:2000 (1% milk in TBST)	One hour (Room temperature)
Anti-Ablim2	Rabbit	Sigma (HPA035808)	1:500 (5% BSA in TBST)	Overnight (4° C)
Anti-STARS	Rabbit	Proteintech (22673-1-AP)	1:1000 (1% milk in TBST)	Overnight (4° C)
Anti-LDH	Mouse	SantaCruz (H-10 #sc-133123)	1:1000 (1% milk in TBST)	Overnight (4° C)
Anti-hnRNPA1	Mouse	SantaCruz (4B10 # SC-32301)	1:1000 (1% milk in TBST)	One hour (Room temperature)
Anti-α-Tubulin	Mouse	Sigma (T9026)	1:2000 (1% milk in TBST)	One hour (Room temperature)

*TBST: Tris buffer solution plus 0.1% Tween 20 (pH 7.5).
All membranes were blocked with 5% milk in TBST for one hour at room temperature.
Secondary antibodies were diluted in 1% milk in TBST 1:10,000.

1. Bray, N. L.; Pimentel, H.; Melsted, P.; Pachter, L. Near-optimal probabilistic RNA-seq quantification. Nat. Biotechnol. 2016, 34, 525–527, doi:10.1038/nbt.3519.

2. Adams Babraham Bioinformatics - FastQC A Quality Control tool for High Throughput Sequence Data Available online: http://www.bioinformatics.babraham.ac.uk/projects/fastqc/ (accessed on Aug 9, 2020).

3. Soneson, C.; Love, M. I.; Robinson, M. D. Differential analyses for RNA-seq: transcript-level estimates improve gene-level inferences. F1000Research 2016, 4, 1521, doi:10.12688/f1000research.7563.2.

4. Love, M. I.; Huber, W.; Anders, S. Moderated estimation of fold change and dispersion for RNA-seq data with DESeq2. Genome Biol. 2014, 15, 550, doi:10.1186/s13059-014-0550-8.

5. Blighe, K.; Rana, S.; Lewis, M. EnhancedVolcano: Publication-ready volcano plots with enhanced colouring and labelin. R-Package 2019.

Uncropped Gels

Related to Figure 5A

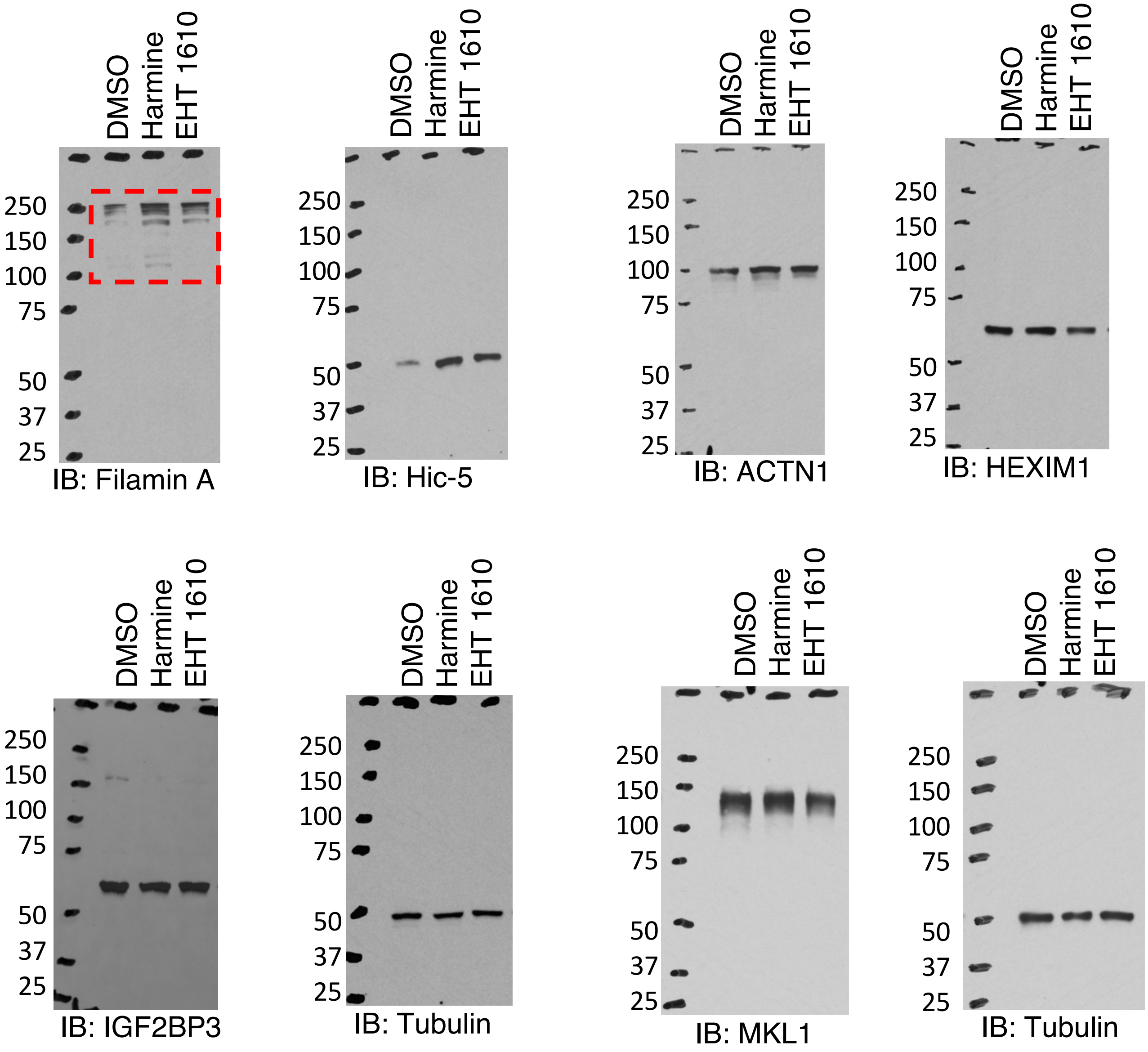

Related to Figure 6C

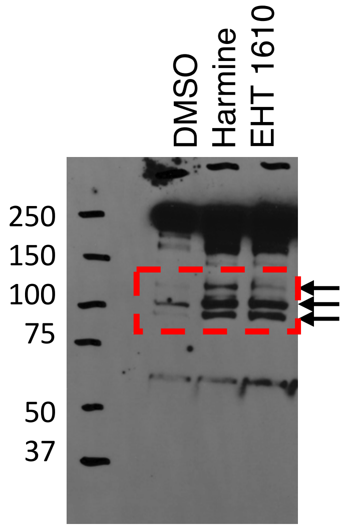

IB: Ablim2

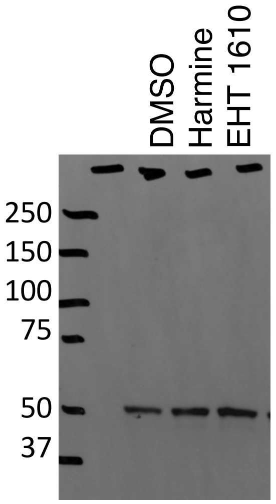

IB: Tubulin

Related to Supplemental Figure 7A

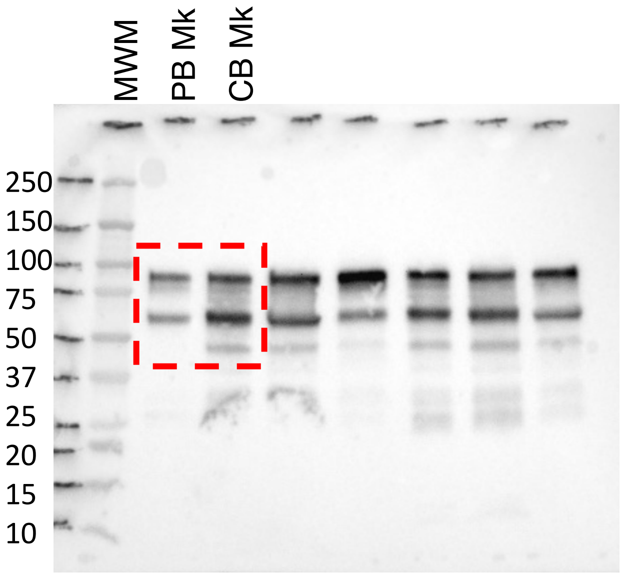

IB: Dyrk1a

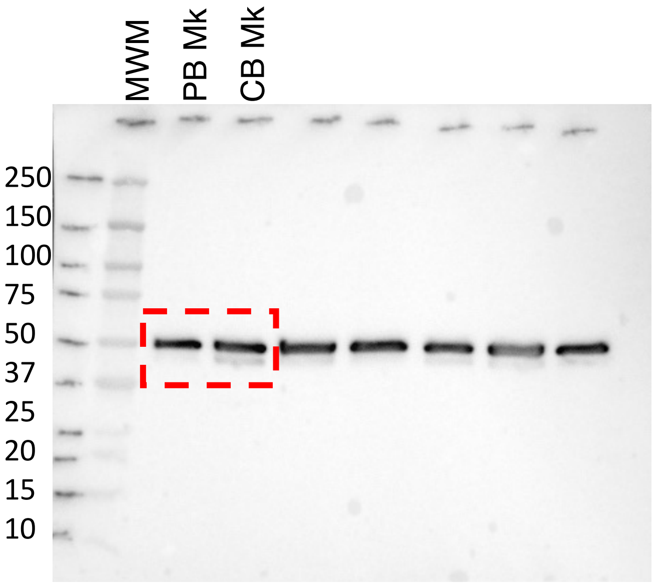

IB: Tubulin

Related to Supplemental Figure 8A

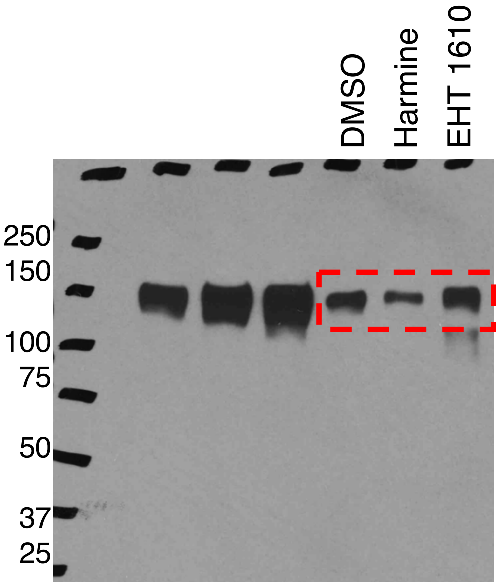

IB: MKL1

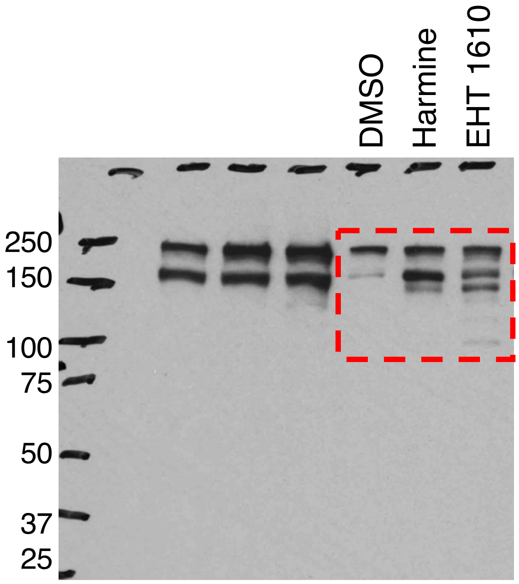

IB: Filamin

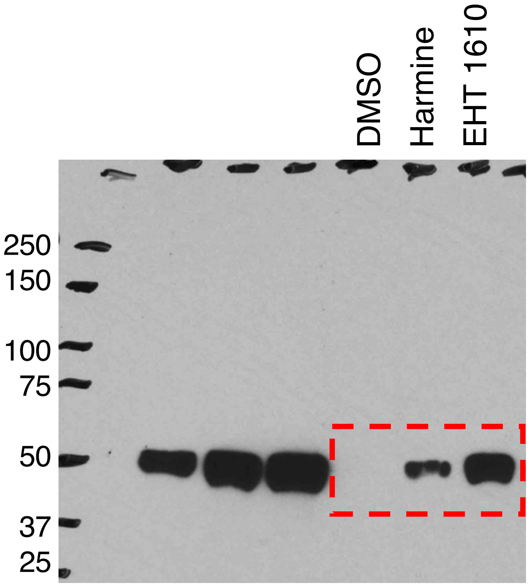

IB: Hic-5

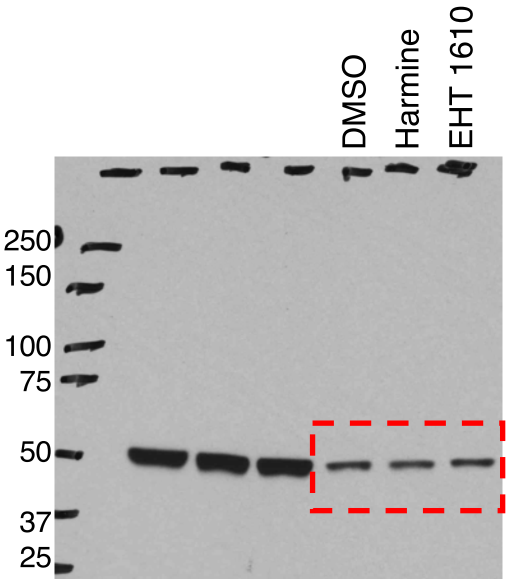

IB: Tubulin

Related to Supplemental Figure 8D

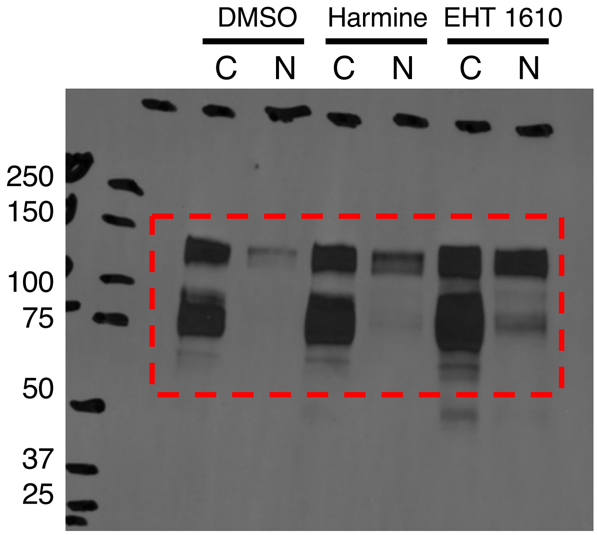

IB: MKL1

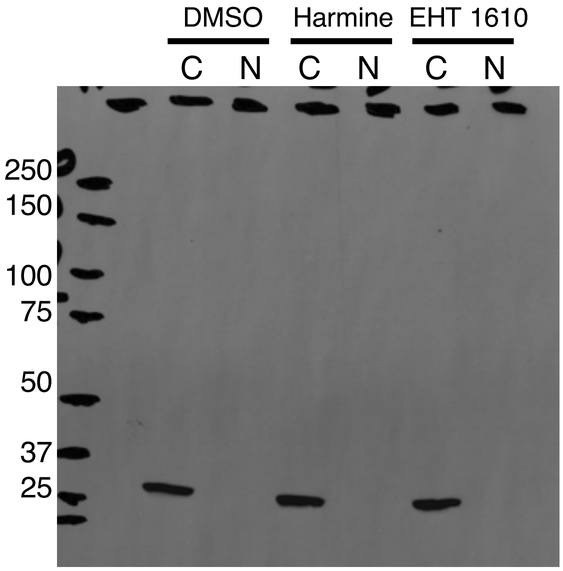

IB: LDH

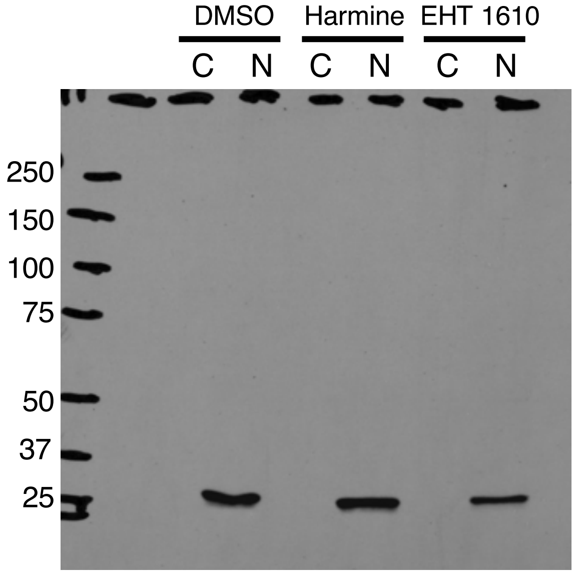

IB: hnRNPA1

C: Cytoplasmic
N: Nuclear

Related to Supplemental Figure 9C

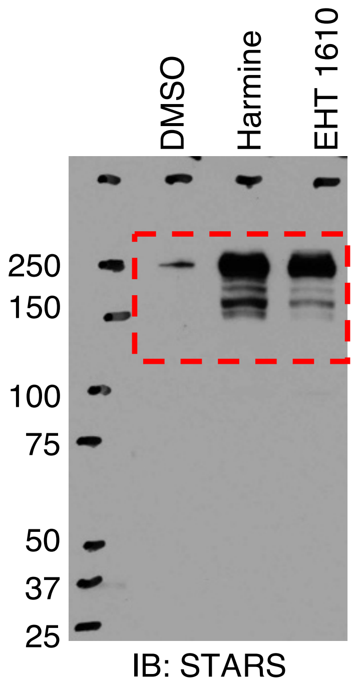

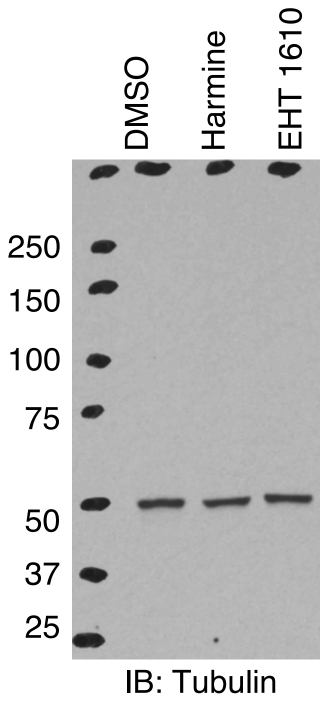

Related to Supplemental Figure 10A

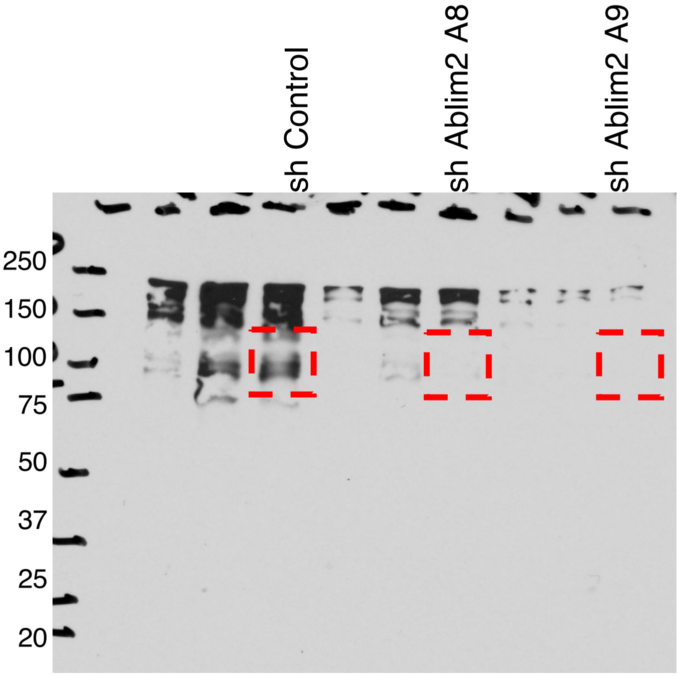

IB: Ablim2

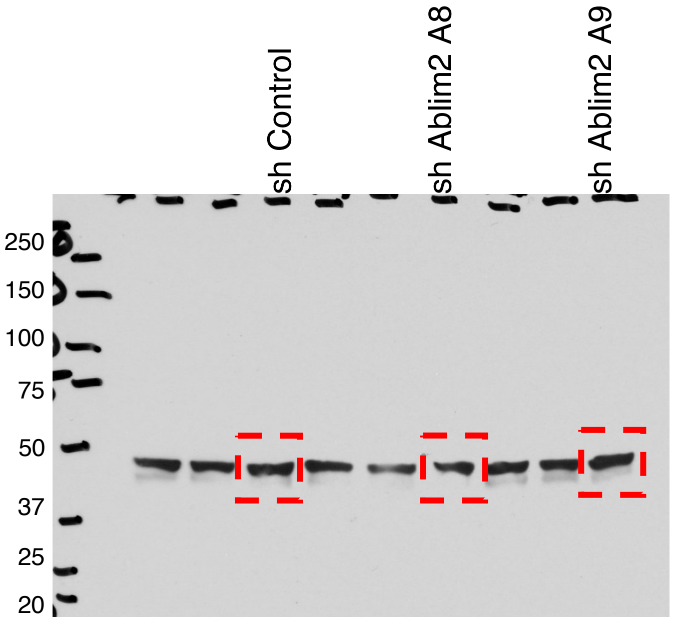

IB: Tubulin

Related to Supplemental Figure 10B

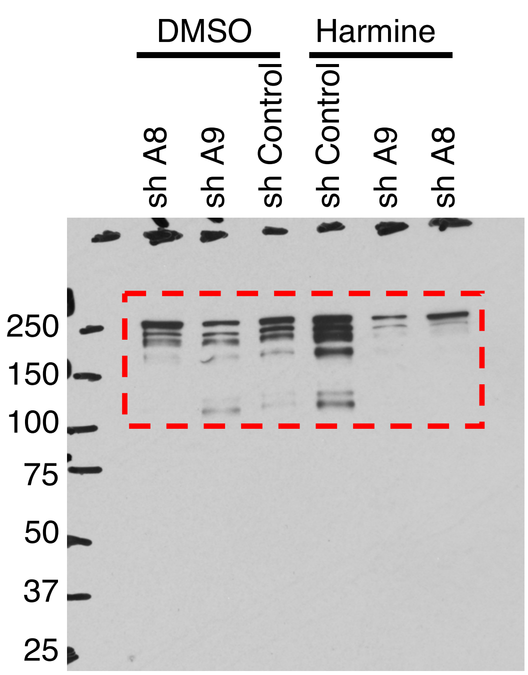

IB: Filamin A

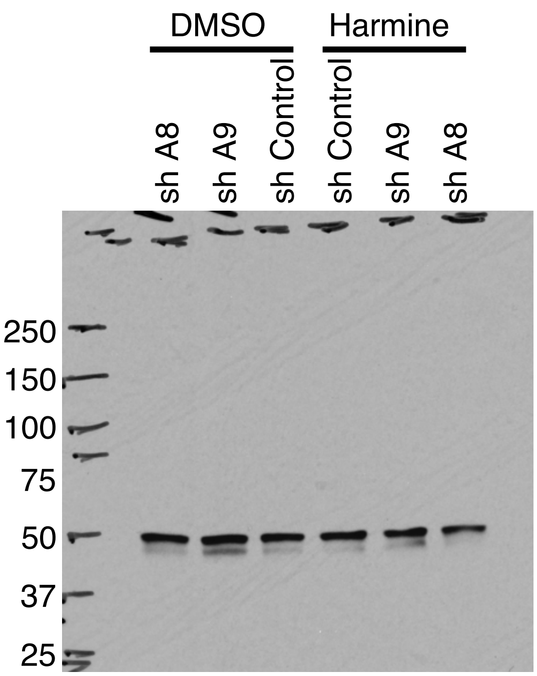

IB: Tubulin
